# Identification and Characterisation of *Pseudomonas savastanoi* pv. *savastanoi* as the Causal Agent of Olive Knot Disease in Croatian, Slovenian and Portuguese Olive (*Olea europaea* L.) Orchards

**DOI:** 10.3390/plants12020307

**Published:** 2023-01-09

**Authors:** Laura Košćak, Janja Lamovšek, Edyta Đermić, Stefania Tegli, Igor Gruntar, Sara Godena

**Affiliations:** 1 Institute of Agriculture and Tourism, Carlo Hugues 8, 52440 Poreč, Croatia; 2 Agricultural Institute of Slovenia, Hacquetova ulica 17, 1000 Ljubljana, Slovenia; 3 Faculty of Agriculture, University of Zagreb, Zagreb, Svetošimunska cesta 25, 10000 Zagreb, Croatia; 4 Laboratorio di Patologia Vegetale Molecolare, Dipartimento di Scienze e Tecnologie Agrarie, Università degli Studi di Firenze, Alimentari, Ambientali e Forestali, Via della Lastruccia 10, 50019 Sesto Fiorentino, Firenze, Italy; 5 Institute of Microbiology and Parasitology, Veterinary Faculty, University of Ljubljana, Gerbičeva 60, 1000 Ljubljana, Slovenia

**Keywords:** LOPAT, colony morphology, strain, molecular identification, MALDI–TOF, olive variety

## Abstract

Strains of *Pseudomonas savastanoi* pv. *savastanoi* (*Pss*), isolated from infected olive trees (*Olea europaea* L.) in three European countries (Croatia, Slovenia and Portugal) were identified and characterised according to their colony morphology, physiological and biochemical features. According to the LOPAT scheme, 38.6% of *Pss* isolates were grouped in the Ib cluster. The Portuguese *Pss* strains were fully consistent with the typical LOPAT profile for this bacterium. Conversely, most Slovenian *Pss* strains showed delayed oxidase activity, whilst Croatian *Pss* strains did not produce any fluorescent pigment when grown in vitro. For *Pss* molecular identification, both end-point and real-time PCR were used, as well as MALDI–TOF, which was additionally used for proteomic analysis and the subsequent species identification of a number of strains that showed deviations from expected LOPAT results. *Pss* was confirmed as a causal agent of olive knot disease in 46.6% of olive orchards screened. Overall, these data suggests a possible correlation of certain *Pss* features with the geographical origin and the ecological niche of *Pss* isolates.

## 1. Introduction

*Pseudomonas syringae* (*P. syringae*) is a species complex of bacteria currently known to include at least 15 species, 13 phylogroups, and more than 60 pathovars [1,2,3]. Most of the strains belonging to the *P. syringae* species complex are plant pathogens or were originally detected in the agro-environment; however, several strains have also been isolated from habitats unrelated to agriculture [4]. *Pseudomonas savastanoi* (*P. savastanoi*) is one of the plant pathogenic species belonging to the *P. syringae* complex and has established infections in a wide array of wild and cultivated plants, including both herbaceous and woody species. However, each pathovar has a specific host range, as expected by definition [5]. The main host of Gammaproteobacterium *P. savastanoi* pv. *savastanoi* (*Pss*) is the olive tree (*Olea europaea* L.). Olives trees are grown in a large area worldwide, and are a typical feature of Mediterranean countries such as Portugal, Croatia and Slovenia, whose productions account for around 722, 150 and 33 thousand tons of olives per year, respectively [6]. Olive oil is important for its benefits to human health, due to its high content of secondary plant metabolites and antioxidants [7]. Olive trees are susceptible to many abiotic and biotic stresses that negatively impact yield and the organoleptic and/or biochemical traits of fruit [8]. In particular, olive knot disease causes significant economic losses in all olive-growing regions [9]. Although the existence of olive knot disease has been known for centuries (mentioned as far back as the 4th century BC by Theophrastus), it is still one of the most challenging plant diseases to control.

Multiple studies on the pathogenicity mechanisms of *P. savastanoi* on herbaceous hosts are available, dating back to [10], whilst in more recent times, data have been collected on the interaction with its woody hosts [11].

In order to develop highly targeted control methods against this bacterium, more information on the pathogenicity and virulence mechanisms used by *P. savastanoi* to attack its hosts is needed. Currently, the analysis of *P. savastanoi* pathogenesis focuses primarily on the requirement of a functional Type Three Secretion System (TTSS) to cause knot formation. This process also involves the bacterial synthesis of indole-3-acetic acid and cytokinin and is influenced by other biological mechanisms, such as quorum-sensing, the synthesis of cyclic -di-GMP and Ca^2+^ cellular homeostasis [5,12,13,14,15,16,17]. For a considerable amount of time, the unequivocal identification of *P. savastanoi* pathovars has been a challenging issue. Previously, taxonomic keys for *P. savastanoi* and other *P. syringae* species and pathovars were somewhat unreliable, and the main approach adopted was based on the evaluation of hosts exhibiting symptoms. Later, bacterial identification and characterisation based on several biochemical and physiological tests, such as the LOPAT scheme became available. The LOPAT scheme consists of a series of determinative tests: L, levan production; O, presence of oxidase activity; P, pectolytic activity; A, arginine dihydrolase activity; and T, tobacco hypersensitivity. This approach is still in use for the identification of phytopathogenic *Pseudomonas* bacteria. 

In accordance with the LOPAT scheme, olive strains of *P. savastanoi* are negative for levan and oxidase production, pectolytic activity, and arginine dihydrolase production and positive for the hypersensitivity reaction in tobacco leaves, and the bacterium was thus categorised into group Ib [18]. However, more recent studies have suggested that this grouping is questionable [19,20,21,22].

Molecular identification methods have remarkably improved in recent decades, enabling more accurate pathogen characterisation and determination. The PCR-based method specific for the causal agent of olive knot disease was developed in the year 2000, using primers for the *iaa*L gene [23], which has subsequently been shown to occur in the genomes of all *P. savastanoi* pathovars. More recently, a number of versatile tools for the in vitro and in planta pathovar differentiation of *P. savastanoi* have been developed, using pathovar-specific probes and High-Resolution Melting Analysis (HRMA). Therefore, there are now molecular diagnostic strategies available that can distinguish *Pss* from *P. savastanoi* pv. *nerii* (*Psn*) and from *P. savastanoi* pv. *fraxini* (*Psf*) [24,25]. This has had a positive impact on disease control strategies enabling the successful identification of pathogens present in symptomatic and asymptomatic plants in nurseries, for example. Another step still to be achieved is related to intra-pathovar genetic differentiation. *P. savastanoi* and *P. syringae* strains and pathovars attacking woody hosts are well segregated from those infecting herbaceous plants [26,27]. Their intra-pathovar and intra-population genetic diversity is generally determined by their geographical origin and by the hosts [28].

Considering the major impact that olive knot disease has on olive production, *Pss* isolates from some Mediterranean regions—even those with a significant olive-growing industry—have been surprisingly poorly studied in terms of biochemical characterisation and molecular identification. Therefore, the main aims of this study were to evaluate the presence of olive knot disease symptoms on various olive varieties in Croatia, Slovenia and Portugal and to characterise the associated *Pss* isolates using the established LOPAT scheme together with relatively newer molecular tests, such as PCR, real-time PCR and MALDI–TOF.

## 2. Results

### 2.1. Distribution and Incidence of Olive Knot Disease Symptoms in Surveyed Countries

More than 600 hectares covering 58 different olive orchards were surveyed, with a total of 206 samples collected. The incidence of olive knot disease on various olive varieties grown in the surveyed orchards is presented in Table 1.

In Croatia, the incidence of olive knot disease was observed on the most prevalent olive varieties, predominantly represented by the most commonly grown olive varieties: Leccino, Pendolino and Istarska bjelica. Moreover, an incidence of 100% of olive knot disease was observed on Nocciara, Italian rosinjola, Buža momjanska, Karbonera, Moražola, Cipressino, Pendolino piangente, Bianchera di Udine and Arbequina varieties, which are grown to a lesser extent, in approximately 3% of olive orchards. Some autochthonous Croatian varieties, such as Buža and Istarska bjelica, were present in more than half of all surveyed areas, and the incidence of olive knot disease was 32% and 30% in the surveyed orchards. Although grown in 13% of sampled orchards, the olive variety Karbonaca exhibited around 83% of disease incidence.

In Slovenian surveyed orchards, the most abundant variety was Istarska bjelica, grown in 75% of orchards. Introduced Italian varieties Leccino and Frantoio were grown in fewer than half of the surveyed orchards, and the incidence of symptoms of olive knot disease was approximately 66% and 100%, respectively. Olive varieties Ascolana tenera, Arbequina, Itrana, Mata, Leccione and Pendolino were grown in 12.5% of orchards. For these varieties, olive knot disease symptoms were found on Mata, Itrana, Leccione, Pendolino, Arbequina and Ascolana tenera, whilst no symptoms were ever observed on the Istarska bjelica variety.

In Portuguese olive orchards, the most prevalent symptomatic variety was Verdeal (60% of sampled orchards), followed by Cobrançosa and Cordovil in 40% of surveyed orchards. The next more prevalent varieties (present in 20% of olive orchards) with symptoms of olive knot disease were: Negruxa de Coimbra, Negruxa, Madura, Negruxa de Tras os Montes and Xuinha/Azeitona de Coimbra. All these varieties in all surveyed Portuguese olive orchards were sampled due to the presence of the typical olive knot disease symptoms.

Overall, the most severe infections were observed on the olive varieties Frantoio, Arbequina, Maurino and Pendolino. Symptoms varied according to the average tumour diameter. In Croatia, Slovenia and Portugal, the largest average tumour diameters were observed on infected plants of the Picholine (1.40 ± 0.20 cm), Mata (3.00 ± 0.00 cm) and Madural (1.03 ± 0.13 cm) varieties, respectively. The lowest values of average knot diameter in Croatia, Slovenia and Portugal were observed on the Buža momjanska (0.60 ± 0.10 cm), Ascolana tenera (0.75 ± 0.25 cm) and Cordovil (0.80 ± 0.00 cm) varieties. The absence of olive knot disease was determined in the Ascolana tenera variety in Croatia and the Istarska bjelica variety in Slovenia.

### 2.2. Biochemical (LOPAT), Physiological and Molecular Tests for Characterisation of Pss Isolates from Infected Olive Materials

*Pss* isolates and their corresponding geographical origins are listed in Table 2. Out of 206 sampled knots collected in 2021 and 2022, in 88 (i.e., 42.7%), the associated bacteria were identified as *Pss* using a real-time PCR method or a standard PCR assay (Figures S1 and S2). For *Pss* isolates from Slovenia, the positive PCR results were confirmed by a mass spectrometry method (MALDI–TOF) (Supplementary S1). The pathogen was thus confirmed to be present and infective in 27 (46.6%) out of a total of 58 olive orchards surveyed in Slovenia, Croatia and Portugal (Table 2).

When tested using a real-time PCR assay, 32 out of 102 (31.4%) and 2 out of 40 (5%) of the bacterial isolates from infected materials from Croatia and Portugal, respectively, were confirmed to be *Pss* (Figure S1). The presence of *Pss* was also confirmed in varieties Buža, Pendolino, Karbonaca, Leccino, Italian rosinjola, Nocciara, Frantoio, Maurino, Porečka rosulja and two unknown varieties in Croatian orchards. On infected plant material from Portugal, *Pss* was isolated only from knots that had developed on Madural and Cordovil varieties. Other cultivars from Portugal exhibited typical *Pss* symptoms but bacterial isolation was unsuccessful due to the advanced ageing of the knots.

A total of 54 out of 60 (90%) bacterial isolates from Slovenia were identified as *P. savastanoi* by PCR (Figure S2). The six isolates that tested negative for *Pss* came from the Itrana variety and were subsequently identified as belonging to non-pathogenic species of *Curtobacterium flaccumfaciens* (Hedges) Collins and Jones and *Bacillus megaterium* de Bary (Supplementary S1 and Table S1). 

**Figure 1:**
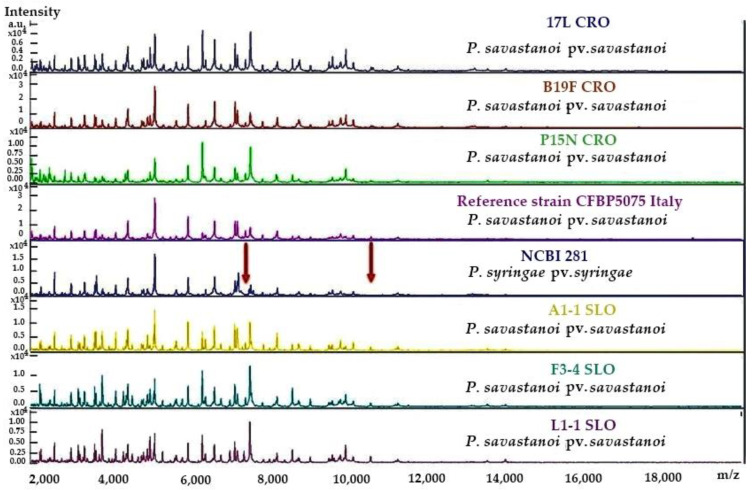
Example MALDI–TOF MS spectra of the six *Pss* strains described in this study and their comparison to *Pss* and *P*. *syringae* pv. *syringae* reference strains’ spectra. The positions of two small protein peaks at approximately 7500–8000 *m/z* and 11,000 *m/z* that are lacking in *P*. *syringae* pv. *Syringae* are indicated by arrows. Axis x: mass per charge in Daltons (*m/z*, Da); y axis: absolute intensity of the signal.

The MALDI–TOF spectra derived from three Croatian *Pss* strains isolated from olive varieties Leccino (17L), Frantoio (B19F) and Nocciara (P15N) and *Pss* Slovenian strains isolated from knots on varieties Arbequina (A1-1), Frantoio (F3-4) and Leccino (L1-1) were selected for analysis due to variabilities in the LOPAT scheme test results for these strains (Figure S3). These strains were homogeneous and clearly identified as *Pss* by the Bruker software, even though the protein profile of *P*. *syringae* pv. *syringae*—the closest genetic relative—was very similar. Typical spectra of some local *Pss* isolates, along with spectra originating from the *Pss* reference strain and from *P*. *syringae* pv. *syringae,* are shown in Figure 1, revealing the uniformity of *Pss* strains and their close resemblance to the *P*. *syringae* pv. *syringae* spectrum. Two small protein peaks—corresponding to approximately 7500–8000 *m/z*, and between 10,000 to 11,000 *m/z*—in *Pss* isolate samples were lacking in the *P*. *syringae* pv. *syringae* strain spectra.

**Figure 2:**
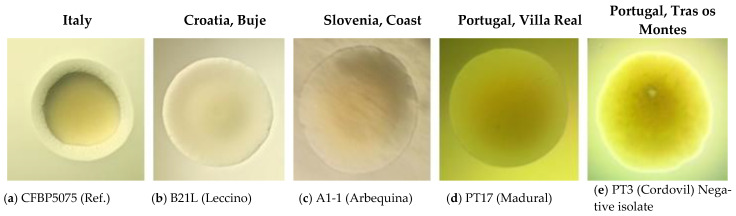
Colony morphology on King’s B (KB) solid growth media of *Pseudomonas savastanoi* pv. *savastanoi* reference strain CFBP5075 (Italy) and strains from Croatian, Slovenian and Portuguese infected olive cultivars: (**a**) CFBP5075 (reference); (**b**) B21 L (Leccino); (**c**) A1-1 (Arbequina); (**d**) PT17 (Madural); (**e**) PT3 (Cordovil). *Pss* colonies presented in figure were tested using the LOPAT scheme, with the following results: levan production: (**a**) negative, (**b**) negative, (**c**) negative, (**d**) negative, (**e**) negative; oxidase activity: (**a**) negative, (**b**) negative, (**c**) delayed positive, (**d**) negative, (**e**) positive; pectolytic activity: (**a**) negative, (**b**) negative, (**c**) negative, (**d**) negative, (**e**) positive; arginine dihydrolase activity—(a) negative, (**b**) negative, (**c**) negative, (**d**) negative, (**e**) negative; tobacco hypersensitivity reaction (HR): (**a**) positive, (**b**) positive, (**c**) positive, (**d**) positive, (**e**) not tested.

In Figure 2, a comparison is shown between the colony morphology o the CFPB5075 strain—used here as a reference for *Pss* grown on King’ B (KB) agarised medium—and of representative *Pss* isolates from Croatia, Slovenia and Portugal. All isolates exhibited similar colony morphology, with a mostly rounded or slightly oval shape. Margins were smooth or corrugated and brighter than the colony centre. Isolates of *Pss* formed colonies with pale white to yellow pigment on KB solid medium. In particular, colonies from Croatian and Slovenian strains were mostly white–greyish, whilst those from Portuguese strains had a yellowish pigmentation. Colony size ranged from 0.5 to 3.0 mm in diameter. Most *Pss* strains from Slovenia formed colonies of 0.5 to 1.0 mm in diameter after three days of incubation on KB. The diameter of *Pss* isolates from Croatia ranged from 1.0 to 3.0 mm, whilst those of Portuguese *Pss* isolates ranged from 1.0 to 2.0 mm after two days of growth (Figure S4).

In the biochemical characterisation, 34 out of 88 (38.6%) isolates tested negative for oxidase, pectolytic activity and the presence of arginine dihydrolase (Table 3 and Table S2). Based on these results, these isolates were classified as group Ib, as expected [29]. The exceptions were Slovenian isolates, which were generally delayed positive for oxidase activity. Only four Slovenian isolates (i.e., 13.3% of the total Slovenian strains coded as L6-2, L5-1, F4-2 and At2-4) were negative for oxidase activity. Most Croatian isolates did not produce fluorescent pigment under UV light when grown on KB solid media. Conversely, all isolates from Slovenia and Portugal tested positive for the in vitro production of fluorescent pigments when grown on solid KB medium.

## 3. Discussion

The field research described in this study included data collected from over 600 hectares of olive orchards surveyed for olive knot disease in Croatia, Slovenia and Portugal. Interestingly, variety Istarska bjelica from Slovenian orchards did not show symptoms of olive knot disease, whereas in Croatia, symptoms were observed on this variety. This observation suggests that the *Pss* infection of Istarska bjelica variety in Slovenia is somehow suppressed.

Notably, higher total phenolic content—considered a major defense mechanism against *Pss*—has been observed in different plant tissues of Istarska bjelica compared to other olive varieties [30,31]. In a recent study of 21 different varieties, all showed a degree of susceptibility to olive knot disease [32], although Istarska bjelica was not included. Interestingly, the variety Itrana in Slovenian orchards showed symptoms of olive knot disease, but the presence of *Pss* was not detected by PCR. The only non-pathogenic bacteria identified by MALDI–TOF were *C. flaccumfaciens* and *B. megaterium*. The effects of the co-infection of these bacterial species with *Pss* needs to be investigated to determine whether they exert a suppressive effect on *Pss* growth in infected olive trees [33,34]. Whilst typical symptoms of olive knot disease were observed in all surveyed Portuguese orchards, *Pss* as the causal pathogen was confirmed only in two samples. However, the isolation of bacteria from these samples was difficult due to the severe aging and cracking of the knots. Despite the challenges, this observation raises the question of whether antagonistic organisms in the surveyed Portuguese region may have infected those trees subsequent to *Pss* infection and consequently suppressed further proliferation of *Pss*. The presence of *Pss* has been detected in Portuguese orchards that also harbour various bacterial species identified as antagonists of *Pss* [34], which may contribute to the suppression of *Pss* growth in the cases we have described.

The epidemiology of olive knot disease in other significant olive-growing countries such as Croatia and Slovenia has been poorly studied up to now. In Slovenia, the first molecular identification of *Pss* was reported in 2016 in symptomatic varieties Leccino, Maurino, Pendolino, Frantoio and Ascolana tenera [35], whilst the first detailed molecular characterisation of a Croatian strain was reported in 2019 [36]. The latter study determined the whole genome sequence of *Pseudomonas* sp. strain ST1 (belonging to *P. amygdali* species), isolated from olive knots from Croatia [33,36]. However, there remains a lack of LOPAT characterisation data on *Pss* that inhabit the olive orchards of Croatia, Slovenia and Portugal, creating a need to evaluate whether such *Pss* isolates have biochemical profiles consistent with pathogenic, tumour-inducing olive-plant bacterial species.

The isolation of bacteria from knots, together with their molecular identification, confirmed that these symptoms are caused by *Pss* in 46.6% of the sampled orchards. Negative results for the presence of *Pss* in sampled plant materials might be caused by aged, dry and cracked knots or may be due to the absence of *Pss* in orchards that have plants displaying symptoms of olive knot disease from prior infections. All isolated *Pss* strains included in this study originate from olive-growing micro-regions, so the biochemical similarities between them are expected, as was observed in the case of levan-positive strains from a limited population distribution in Italy, for example [21,22]. However, some differential characteristics can be determined within micro-regions (even between strains isolated from the same plant [29,37]), similar to what was observed in the present study. For molecularly confirmed *Pss* isolates, LOPAT results were recorded that deviated from those expected for segregated strains. Additionally, differences between *Pss* strains isolated from the same orchard were noted. All isolates from Portugal (coded as PT15 and PT17) showed typical LOPAT results for olive strains [37]. *Pss* formed mostly round or slightly oval shaped colonies, with white–greyish to yellowish pigmentation, with a diameter range from 0.5 to 3 mm. However, some variability in *Pss* colony morphology was observed (Figure S4), which has previously been ascribed to adaptation to different environmental conditions or horizontal gene transfer [37,38].

Although the differences in the phenotype of *Pss* colonies were expected, the results of the LOPAT biochemical identification of *Pss* isolates showed a clear separation, or even grouping, of most Slovenian strains. The majority of Slovenian strains exhibited delayed positive oxidase activity. Another grouping, which contained most of the Croatian isolates, was characterised as having a lower occurrence of fluorescent pigment on solid KB medium in vitro. However, the mass spectroscopy analysis showed high similarities in the protein profiles of selected Slovenian and Croatian strains (Figure 1). These results are interesting, given that *Pss* was confirmed on a single plant species (olive), wherein similarities in biochemical features are to be expected [39]. However, from a genetic point of view, some segregation of strains occurs as a result of different geographical origins or factors acting within a specific ecological niche, which might also occur on a micro-location level. Although the fluorescent pigment was absent in Croatian strains, this feature is considered variable for *Pss* bacteria [21,38,40]. It has been observed that fluorescence does not always occur in so-named `fluorescent’ Pseudomonads [41], and our results support this. Similar results were determined for some Australian and Japanese strains [20,21]. However, it is important to mention that the absence of fluorescent pigment on KB medium usually occurs when strains are levan-positive, which did not occur in our case.

In light of the interesting results obtained in this study, more genetic and phylogenetic research should be done to better understand the pathogen’s virulence in relation to geographical origin and olive cultivars. This study also contributes to the mapping of the geographical distribution of pathovar *Pss*, with the aim to update available databases that still present the distribution of three different pathovars of *P. savastanoi* tumour-inducing bacteria (*Pss*, *Psn* and *Psf*) as one tumour-inducing species. Clear differentiation is obligatory, due to the established differences in the virulence of these woody-host pathogens [15,42]. Furthermore, the study of the susceptibility of Croatian and Slovenian autochthonous varieties to olive knot disease should be examined. These autochthonous olive cultivars could elicit some resistance traits, which are as yet unknown. According to more recent studies, including ours, an update of the LOPAT scheme should be considered, due to deviations from the expected grouping of *Pss* isolates [18,19,20,21,22]. In the literature [42], it is stated that strains of *Pss* originating from the former Yugoslavia region are amongst the least-virulent strains, whilst strains from Portugal are of intermediate pathogenicity, which demonstrates the existence of virulence differences between *Pss* strains according to geographical origin. These observations warrant further examination of the role of geographical and environmental factors of *Pss* strain grouping in the future studies.

## 4. Materials and Methods

### 4.1. Plant Material Sampling

Samples of olive knots from different varieties of symptomatic olive trees present in the Croatian and Slovenian part of Istria, as well as in Portugal, were collected from April to September of 2021 and from March to May of 2022. The surveyed area comprised the northernmost point (Goriška Brda in Slovenia, 46°00′ N; 13°57′ E), the southernmost and westernmost point (Tras-os-Montes, Vila Real, Mesão Frio in Portugal, 41°09′ N; 7°53′ W) and the easternmost point (Pula region in Croatia, 44°89′ N; 13°81′ E) (Table 1). Samples of symptomatic plant material were sealed in plastic bags, correctly labeled and kept in a portable fridge. After field research, samples were stored at +4 °C until processed.

### 4.2. Bacterial Isolation and Characterisation

The bacterial isolation was performed from knots of randomly collected infected olive branches. Olive knots were washed under tap water to remove dust, followed by surface disinfection with 70% ethanol and left to dry. After disinfection, knots were aseptically cut into small pieces and placed into sterile distilled water for 30 min. The resulting suspensions were serially diluted 10-fold, two times. A volume of 100 µL of each sample dilution was placed on King’s B (KB) solid growth media supplemented with cycloheximide (100 mg/mL). The morphology of the obtained colonies was visualised by a Trinocular Microscope BOE 2200.520 (BOECO, Hamburg, Germany) at 4.00× magnification. The biochemical and physiological characterisation of bacterial colonies was carried out according to the LOPAT scheme [18,43]. For this purpose, pure bacterial cultures were spread on growth media. Bacterial cultures were characterised based on fluorescence (366 nm wavelength) on KB, measured after 24–48 h of incubation, Gram staining using 3% potassium hydroxide (KOH) and the LOPAT scheme. A bacterial inoculum of 10^8^ CFU/mL was used for physiological and biochemical tests. For determination of levan production, pure cultures were streaked onto 5% sucrose nutrient agar (SNA) and incubated for 3–5 days for the development of white mucoid colonies in positive isolates. The oxidase reaction test was performed using commercial MAST ID^TM^ Oxidase strips (Mast House, UK) following the manufacturer’s instructions. Pectolytic activity was determined on disinfected potato slices in Petri dishes with moistened filter paper, whilst the presence of arginine dihydrolase was determined using Thornley’s medium 2A with pH adjusted to 7.2. The total volume used was 5 mL. A colour change from pink to red within four days was recorded as a positive reaction. The hypersensitivity reaction was carried out on tobacco variety *Nicotiana tabbacum* L. cv. *Samsun* using syringae without a needle for inoculation with bacterial suspensions. The complete collapse of impregnated leaf tissue within 24 h was recorded as a positive result. The morphological characterisation of bacterial colonies included measuring diameter (mm) after 24 h, shape, pigmentation, margin shape and smoothness. All isolates from Croatia and Portugal (of which there were 35 and 2, respectively) were characterised. For the characterisation of Slovenian isolates, 30 *Pss* strains obtained from different olive varieties were selected at random.

### 4.3. Molecular Identification of Pss

The specific detection of *Pss* was achieved by real-time PCR using TaqMan^®^ probes according to the method of Tegli et al. [24] after growing bacteria on KB and PVF-1 media incubated at 26 °C for 48 h. Bacterial DNA was isolated using a Maxwell^®^ RSC Instrument (Promega, Madison, WI, USA) and Maxwell^®^ RSC Cultured Cells DNA Kit (Promega, Madison, WI, USA), following the manufacturer’s instructions. Primers and probes sequences were as follows: *Psv*RT-F 5′ CGGATTTGGTTTGCGGGGTA 3′, *Psv*RT-R 5′ AATGGGGTGACACTAAAAATTGTGAA 3′, *Psv*RT-P 5′ VIC-CTCGTGCGATCTAAACAGCCGTAGC-QSY 3′.

All obtained isolates and the reference strain were included in this survey. Reference strain CFBP5075 (Italy) was purchased from the National Institute of Agricultural Research, INRA (Paris, France). The PCR reaction was performed in an Applied Biosystems Quant Studio 1 PCR machine (Thermo Fisher Scientific, Waltham, MA, USA). MicroAmp^®^ Optical 96-well reaction plates from Applied Biosystems^®^ (Waltham, MA, USA) were used. The reaction mixture volume was 50 µL using the following reagent concentrations: 1x TaqMan^®^ Universal PCR Master Mix, 300 nM of each primer (*Psv*RT-F and *Psv*RT-R), 100 nM of probes (*Psv*RT-P) and 3 µL of DNA.

For Slovenian isolates, the identification of *Pss* from olive knots was based on a standard PCR assay [22]. Bacterial DNA was extracted from pure cultures by the boiling method. A loop-full of bacteria grown on KB medium for 24 h at 26 °C was suspended in 500 µL of sterile water, heated to 95 °C for 10 min and immediately cooled on ice. Dilutions (1:1000) were prepared in sterile TE buffer. For the amplification of the *iaa*L gene, we used IAALF and IAALR primers designed by Penyalver et al. [23], which produced a 454 bp-long amplified fragment. PCR assays were performed in a final volume of 25 µL in a reaction mixture containing 1x GoTaq PCR buffer (Promega), 1.5 mM MgCl_2_, 125 µM of each dNTP, 0.25 µM of each primer, 1 U of GoTaq Flexi polymerase (Promega) and 2 µL of diluted bacterial DNA. Cycling conditions comprised an initial denaturing step of 5 min at 94 °C; followed by 35 cycles of 94 °C (30 s), 62 °C (30 s) and 72 °C (30 s); and a final elongation step at 72 °C (5 min). Amplified PCR fragments were visualised on 1% agarose gel.

Additional confirmation of the PCR assay results was based on MALDI–TOF MS, conducted by the microbiology laboratory at the Institute of Microbiology and Parasitology (University of Ljubljana, Ljubljana, Slovenia). Analysis was performed on pure 24 h-old cultures of all 60 Slovenian isolates.

### 4.4. Protein Mass Spectra Analysis

Protein profiles of six *Pss* strains from Croatia and Slovenia, and the reference strain CFBP5075 (Italy), were examined by MALDI–TOF mass spectrometry. The analyses were performed on a Bruker Microflex LT mass spectrometer (Bruker Daltonics, Billerica, MA, USA), according to the manufacturer’s instructions and using direct transfer sample preparation with standard α-cyano-4-hydroxycinnamic acid (HCCA) bacterial cell extraction [44]. The obtained spectra were compared to those of the *Pss* reference strain and their closest phylogenetic neighbour, *P. syringae* pv. *syringae*. Briefly, the bacteria were grown on KB medium for 24 h at 28 °C. A small fraction of a single colony from each strain was then transferred to a corresponding spot on the Bruker 96-well target plate and treated with 1 µL HCCA reagent. After plate insertion and following the MALDI–TOF sample processing procedure, 40 sub-spectra for each of the 40 randomised positions within the spot were collected and presented as one main spectrum for each spot/bacterial strain. A mass range of 2000 to 20,000 Da was used for the analysis. The mass spectra profiles were identified from the mass spectra profiles using the Bruker MBT Compass HT software.

## Figures and Tables

**Table 1:** Incidence of olive knot disease on different olive varieties in surveyed Croatian, Slovenian and Portuguese olive orchards.

Surveyed Country	Area (ha)	Olive Varieties (%)	Incidence * (%)	Severity ^§^ (cm)
Croatia	600	Leccino (82)	59.5	0.97 ± 0.22
		Pendolino (69)	48.4	1.04 ± 0.22
		Istarska bjelica (67)	30.0	0.85 ± 0.17
		Frantoio (58)	67.9	1.21 ± 0.33
		Buža (56)	32.0	0.88 ± 0.20
		Maurino (18)	50.0	0.88 ± 0.08
		Picholine (16)	14.3	1.40 ± 0.20
		Moraiolo (13)	33.3	0.75 ± 0.05
		Karbonaca (13)	83.3	0.76 ± 0.08
		Rosinjola (11)	20.0	0.80 ± 0.01
		Leccio del Corno (9)	25.0	1.20 ± 0.40
		Ascolana tenera (9)	0.00	0.00 ± 0.00
		Porečka rosulja (7)	33.3	0.90 ± 0.10
		Italian rosinjola (3)	100	0.80 ± 0.20
		Nocciara (3)	100	0.80 ± 0.10
		Buža momjanska (3)	100	0.60 ± 0.10
		Karbonera (3)	100	1.00 ± 0.20
		Moražola (3)	100	0.80 ± 0.01
		Cipressino (3)	100	0.80 ± 0.01
		Pendolino piangente (3)	100	1.10 ± 0.30
		Bianchera di Udine (3)	100	0.90 ± 0.30
		Arbequina (3)	100	0.70 ± 0.10
		Ascolana tenera (12.5)	100	0.75 ± 0.25
Slovenia	2.40	Frantoio (25) Ascolana tenera (12.5)	100 100	1.97 ± 2.02 0.75 ± 0.25
		Pendolino (12.5) Frantoio (25)	100 100	1.00 ± 0.00 1.97 ± 2.02
		Itrana (12.5) Pendolino (12.5)	100 100	2.80 ± 0.40 1.00 ± 0.00
		Mata (12.5) Itrana (12.5)	100 100	3.00 ± 0.00 2.80 ± 0.40
		Leccione (12.5) Mata (12.5)	100 100	1.00 ± 0.00 3.00 ± 0.00
		Leccino (37.5) Leccione (12.5)	66.6 100	2.14 ± 1.36 1.00 ± 0.00
		Arbequina (25) Leccino (37.5)	50.0 66.6	1.00 ± 0.00 2.14 ± 1.36
		Istarska bjelica (75) Arbequina (25)	00.0 50.0	0.00 ± 0.00 1.00 ± 0.00
		Istarska bjelica (75)	00.0	0.00 ± 0.00
		Cobrançosa (40)	100	0.83 ± 0.14
Portugal	3.25	Cordovil (40) Cobrançosa (40)	100 100	0.80 ± 0.00 0.83 ± 0.14
		Negruxa de Coimbra (20) Cordovil (40)	100 100	0.89 ± 0.08 0.80 ± 0.00
		Negruxa (20) Negruxa de Coimbra (20)	100 100	0.91 ± 0.06 0.89 ± 0.08
		Verdeal (60) Negruxa (20)	100 100	0.95 ± 0.14 0.91 ± 0.06
		Madural (20) Verdeal (60)	100 100	1.03 ± 0.13 0.95 ± 0.14
		Negruxa de Tras os Montes (20) Madural (20)	100 100	0.96 ± 0.19 1.03 ± 0.13
		Xuinha/Azeitona de Coimbra (20) Negruxa de Tras os Montes (20)	100 100	1.00 ± 0.00 0.96 ± 0.19
		Xuinha/Azeitona de Coimbra (20)	100	1.00 ± 0.00

* Number of trees with olive disease symptoms as a percentage of total surveyed trees of the same olive variety. ^§^ Diameter of olive knot (data are presented as mean values with standard deviation of 10 randomly measured knots for each collected sample per variety).

**Table 2:** *Pss* isolates identified in olive varieties from Croatian, Slovenian and Portuguese orchards with the relevant olive orchards’ coordinates and the molecular methods used for pathovar identification.

*Pss* Isolate	Olive Variety	Country, Region, Municipality	Orchard Coordinates	*Pss* Specific Real-Time Assay *	PCR and MALDI–TOF
V4B	Buža	Croatia, Istria, Vodnjan	44°56′99″ N; 13°51′81″ E	+	
V4 P	Pendolino				
V7 K	Karbonaca	Croatia, Istria, Vodnjan	45°57′99″ N; 13°51′81″ E	+	
V9 B	Buža	Croatia, Istria, Vodnjan	44°58′58″ N; 13°49′52″ E	+	
R12 L	Leccino	Croatia, Istria, Rovinj	45°06′12″ N; 13°69′24″ E	+	
P15 TR	Italian rosinjola	Croatia, Istria, Poreč	45°17′12″ N; 13°39′56″ E	+	
P15 N	Nocciara				
B17 L	Leccino	Croatia, Istria, Buje	45°21′40″ N; 13°32′47″ E	+	
B19 F	Frantoio	Croatia, Istria, Buje	45°24′50″ N; 13°35′14″ E	+	
B19 L	Leccino				
B19 P	Pendolino				
B21 L B21F	Leccino Frantoio	Croatia, Istria, Buje	45°22′45″ N; 13°43′38″ E	+	
B23 F	Frantoio	Croatia, Istria, Buje	45°20′20″ N; 13°33′31″ E	+	
I6 L, I7 L	Leccino	Croatia, Istria, Poreč	45°22′34″ N; 13°60′25″ E	+	
NIN	Unknown	Croatia, Istria, Pula	44°89’56’’ N; 13°81’82’’ E	+	
U26 F	Frantoio	Croatia, Istria, Umag	45°24′27″ N; 13°33′24″ E	+	
U27 F	Frantoio	Croatia, Istria, Umag	45°28′43″ N; 13°30′43″ E	+	
VS28 N	Unknown	Croatia, Istria, Višnjan	45°15′58″ N; 13°43′20″ E	+	
VS28 L	Leccino	Croatia, Istria, Višnjan	45°15′58″ N; 13°43′20″ E	+	
VS29 L	Leccino	Croatia, Istria, Višnjan	45°17′53″ N; 13°42′50″ E	+	
VS31 L	Leccino	Croatia, Istria, Višnjan	45°14′44″ N; 13°42′15″ E	+	
R34 F	Frantoio	Croatia, Istria, Rovinj	45°02′53″ N; 13°44′10″ E	+	
BR37 L	Leccino	Croatia, Istria, Brtonigla	45°22′48″ N; 13°36′28″ E	+	
N40 M	Maurino	Croatia, Istria, Novigrad	44°21′46″ N; 13°33′46″ E	+	
N41 F	Frantoio	Croatia, Istria, Novigrad	45°21′34″ N; 13°35′77″ E	+	
N42 F	Frantoio	Croatia, Istria, Novigrad	45°21′06″ N; 13°35′01″ E	+	
N43 L N43 P	Leccino Pendolino	Croatia, Istria, Novigrad	45°22′05″ N; 13°33′40″ E	+	
P44 F	Frantoio	Croatia, Istria, Poreč	45°14′26″ N; 13°42′17″ E	+	
B45 C-PR	Porečka rosulja	Croatia, Istria, Buje	45°24′42″ N; 13°30′10″ E	+	
F1-2, F1-3, F1-5F2-2, F2-5 A1-1, A1-3, A1-4 L1-1, L2-1, L2-3 AT1-2, AT2-6, AT2-4, AT1-5, AT1-4	Frantoio Arbequina Leccino Ascolana tenera	Slovenia, Coast	45°31′59″ N; 13°30′10″ E		+
L6-2, L6-4 L5-3, L5-1, L5-4 L4-1, L4-4 L6-1	Leccino	Slovenia, Goriška Brda	46°0′11″ N; 13°34′36″ E		+
F3-2, F3-4, F3-5 F4-2, F4-1, F4-4	Frantoio	Slovenia, Goriška Brda	45°58′56″ N; 13°30′29″ E		+
PT15 PT 17	Cordovil Madural	Portugal, Villa Real	41°09′41″ N; 7°51′20″ W	+	

* Tegli et al. [24].

**Table 3:** LOPAT characteristics of *P. savastanoi* pv. *savastanoi* isolates from Croatian, Slovenian and Portuguese olive orchards.

Characteristic	Croatia (Istria)	Slovenia (Coast and Goriška Brda)	Portugal (Villa Real)
Gram-reaction	+	+	+
Fluorescence on KB	-	+	+
Levan production	-	-	-
Oxidase reaction	-	DP	-
Pectolytic activity	-	-	-
Arginine dihydrolase activity	-	-	-
Tobacco HR	+	+	+

+ more than 70% of isolates were positive for tested characteristic; - more than 70% of isolates were negative for tested characteristic; DP-delayed positive.

## Data Availability

Not applicable.

## Supplementary Materials

The following supporting information can be downloaded at: https://www.mdpi.com/article/10.3390/plants12020307/s1, Supplementary S1: Slovenian PCR positive *Pss* isolates confirmed using mass spectrometry; Table S1: Bacteria strains isolated from olive knots in Slovenia identified using mass spectrometry; Table S2: Characterisation of molecularly identified *Pss* isolates; Figure S1: Amplification plots of positive *Pss* isolates from Croatia and Portugal. Figure S2: Gel electrophoresis of end-point PCR identified *Pss* isolates from Slovenia. Figure S3: Biochemical and physiological characteristics of *Pss* isolates. Figure S4: Colony morphology of tested *Pss* isolates.
