# Supplementary material for: Identification and Characterisation of Pseudomonas savastanoi pv. savastanoi as the Causal Agent of Olive Knot Disease in Croatian, Slovenian and Portuguese Olive (Olea europaea L.) Orchards
Source: Plants (Basel). 2023 Jan 9;12(2):307. doi: 10.3390/plants12020307 (PMC9865541; doi:10.3390/plants12020307)
Supplement: Supplementary file 1 [file plants-12-00307-s001.zip › Figure S1. Amplification plots of positive Pss isolates from Croatia and Portugal.pdf]

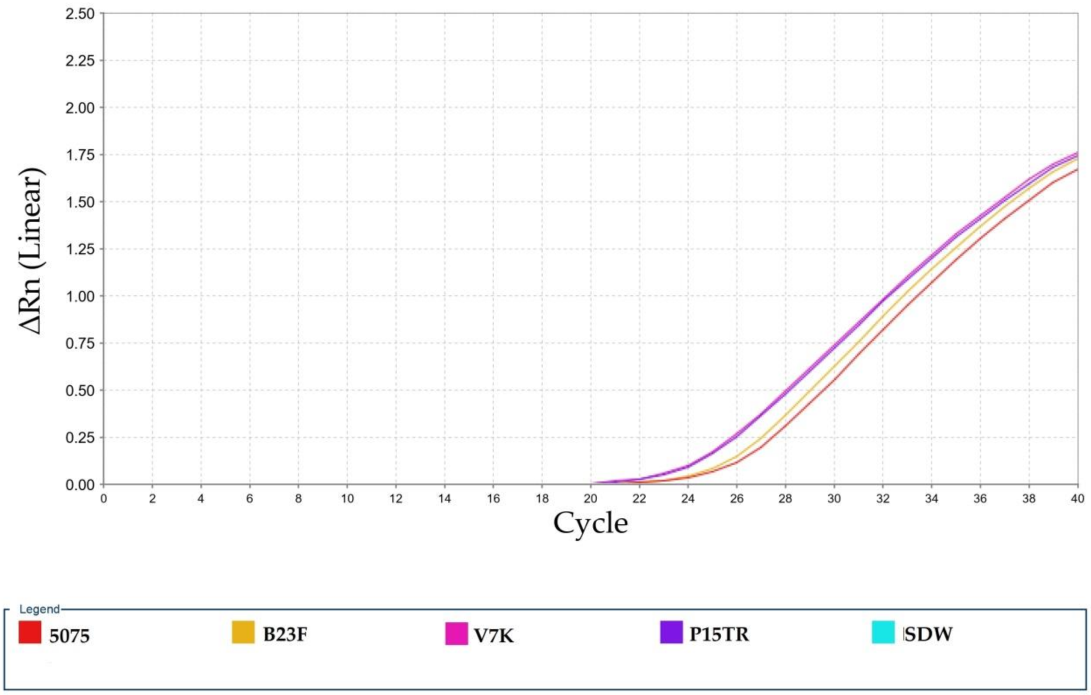
ΔRn (Linear)
Cycle
Legend
5075
B23F
V7K
P15TR
SDW

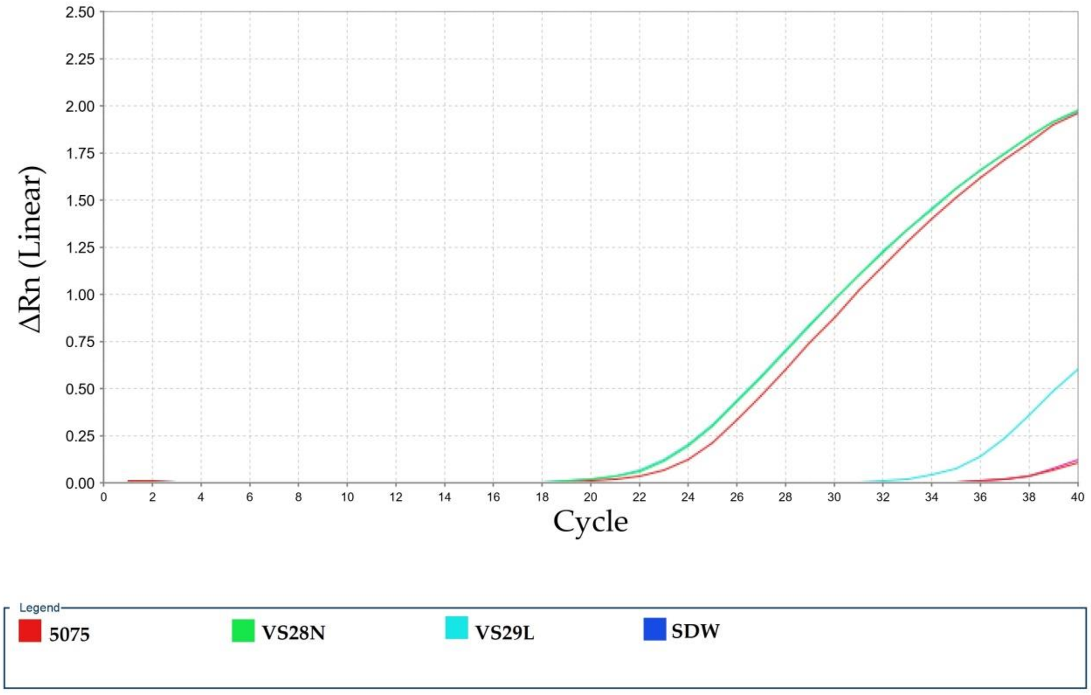
ΔRn (Linear)
Cycle
Legend
5075
VS28N
VS29L
SDW

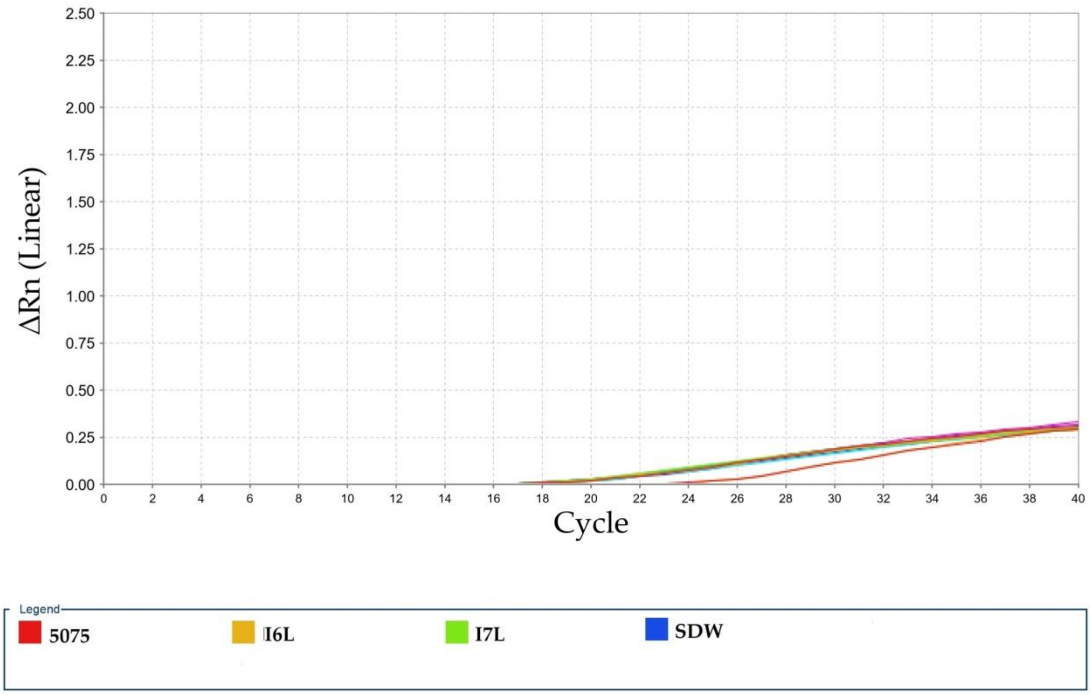
2.50
2.25
2.00
1.75
1.50
1.25
1.00
0.75
0.50
0.25
0.00
ΔRn (Linear)
0
2
4
6
8
10
12
14
16
18
20
22
24
26
28
30
32
34
36
38
40
Cycle
Legend
5075
I6L
I7L
SDW

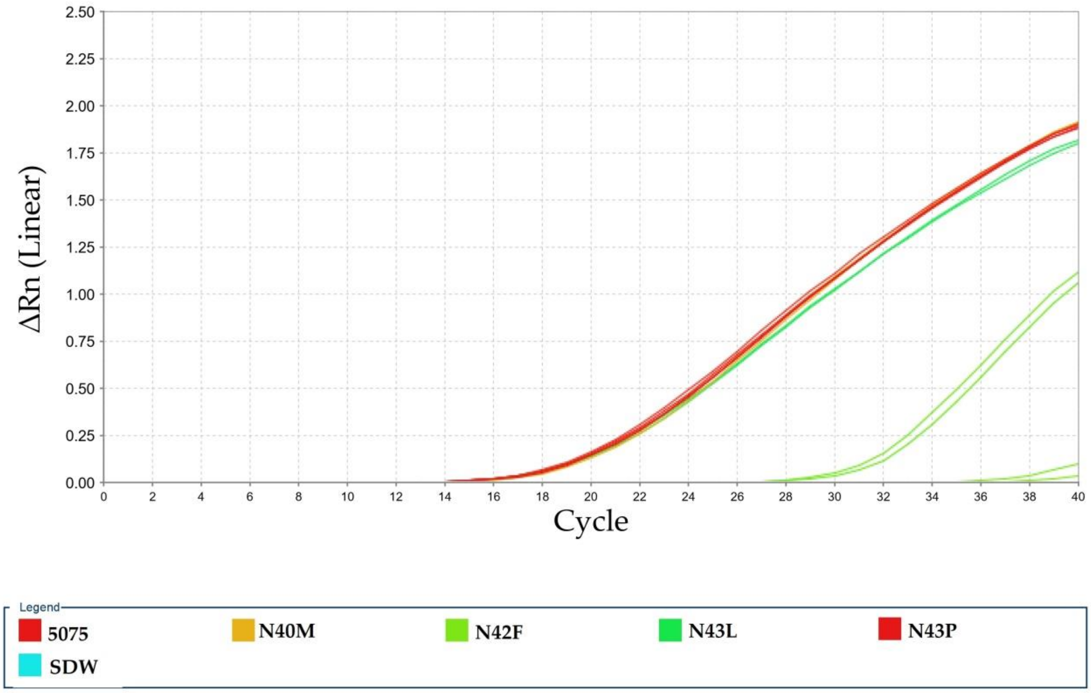
2.50
2.25
2.00
1.75
1.50
1.25
1.00
0.75
0.50
0.25
0.00
ΔRn (Linear)
0
2
4
6
8
10
12
14
16
18
20
22
24
26
28
30
32
34
36
38
40
Cycle
Legend
5075
N40M
N42F
N43L
N43P
SDW

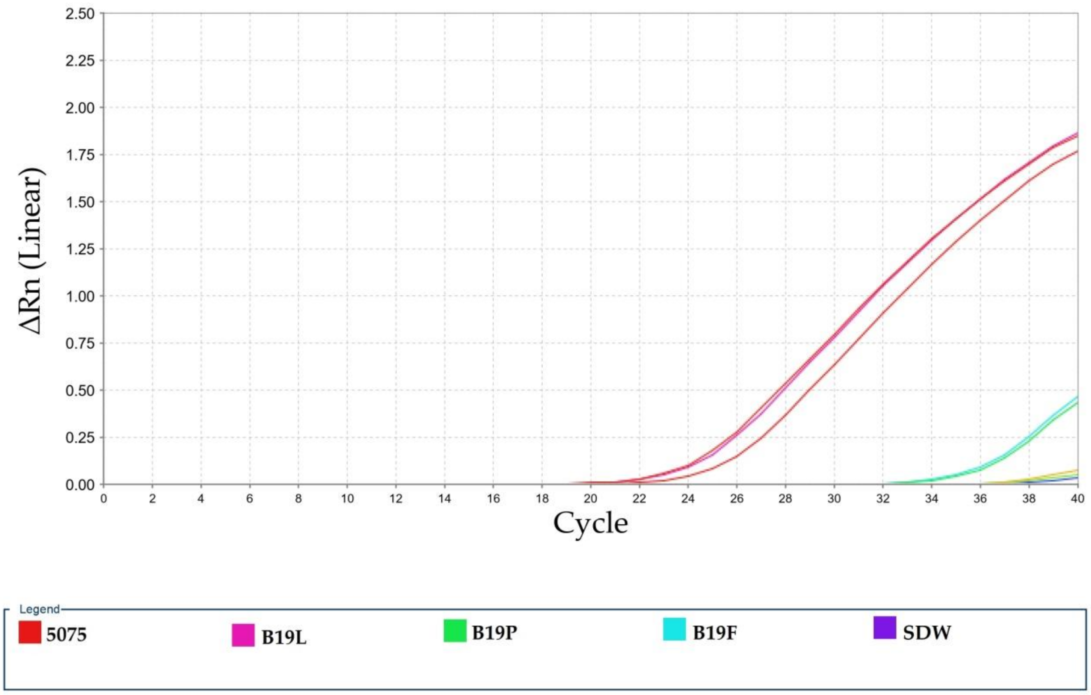
2.50
2.25
2.00
1.75
1.50
1.25
1.00
0.75
0.50
0.25
0.00
ΔRn (Linear)
0 2 4 6 8 10 12 14 16 18 20 22 24 26 28 30 32 34 36 38 40
Cycle
Legend
5075
B19L
B19P
B19F
SDW

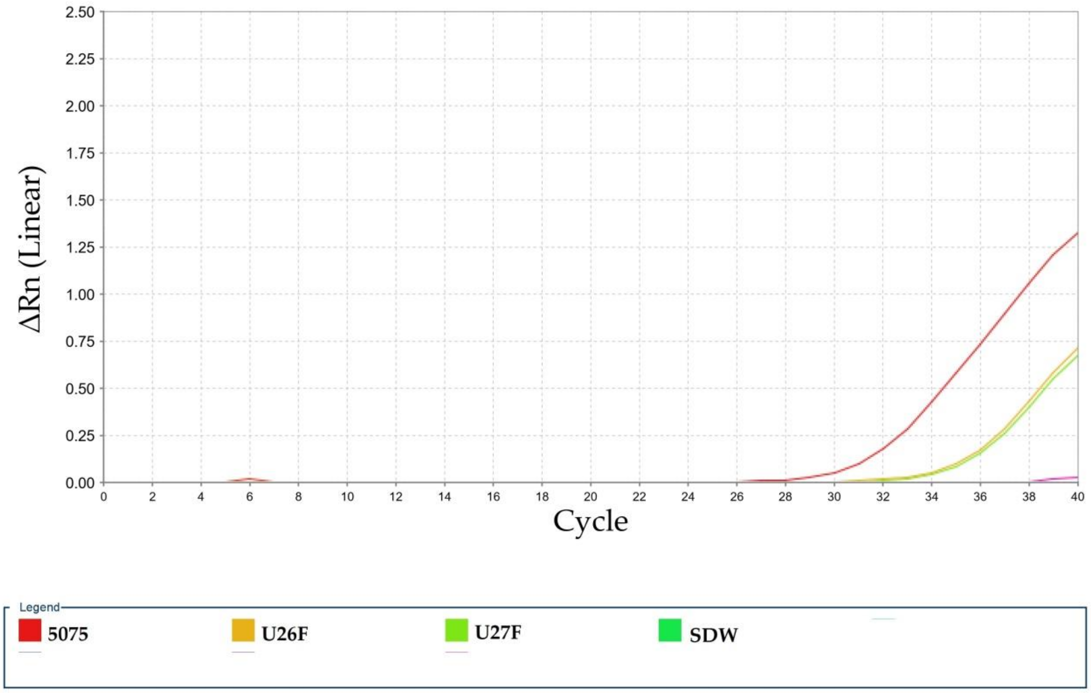
2.50
2.25
2.00
1.75
1.50
1.25
1.00
0.75
0.50
0.25
0.00
ΔRn (Linear)
0 2 4 6 8 10 12 14 16 18 20 22 24 26 28 30 32 34 36 38 40
Cycle
Legend
5075
U26F
U27F
SDW

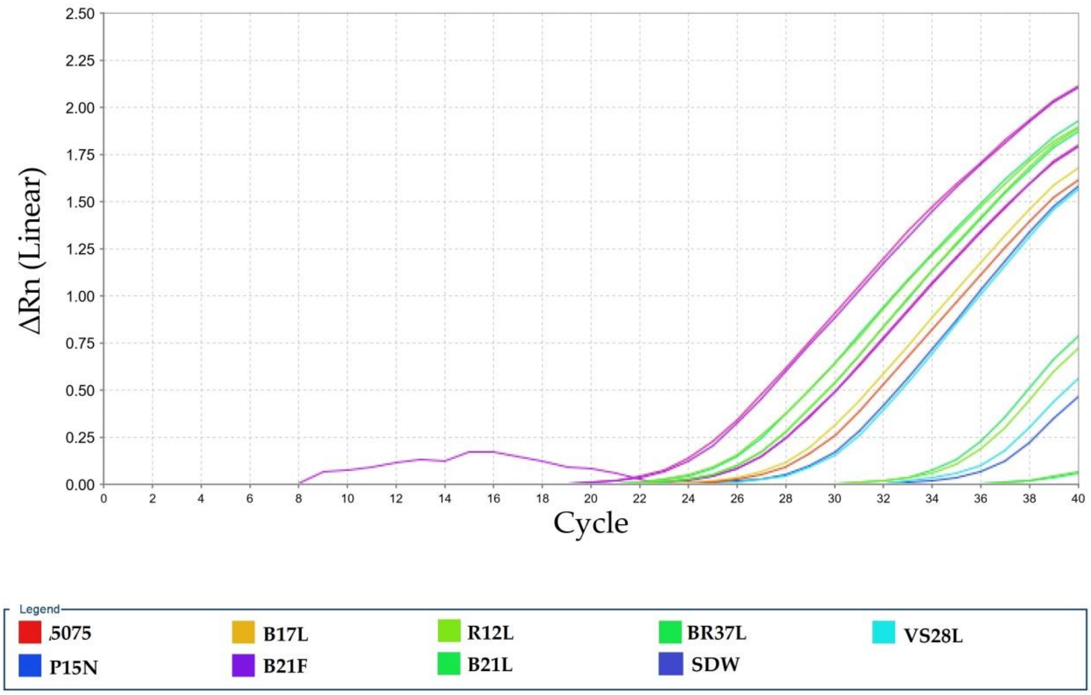
ΔRn (Linear)
2.50
2.25
2.00
1.75
1.50
1.25
1.00
0.75
0.50
0.25
0.00
0 2 4 6 8 10 12 14 16 18 20 22 24 26 28 30 32 34 36 38 40
Cycle
Legend
,5075
B17L
R12L
BR37L
VS28L
P15N
B21F
B21L
SDW

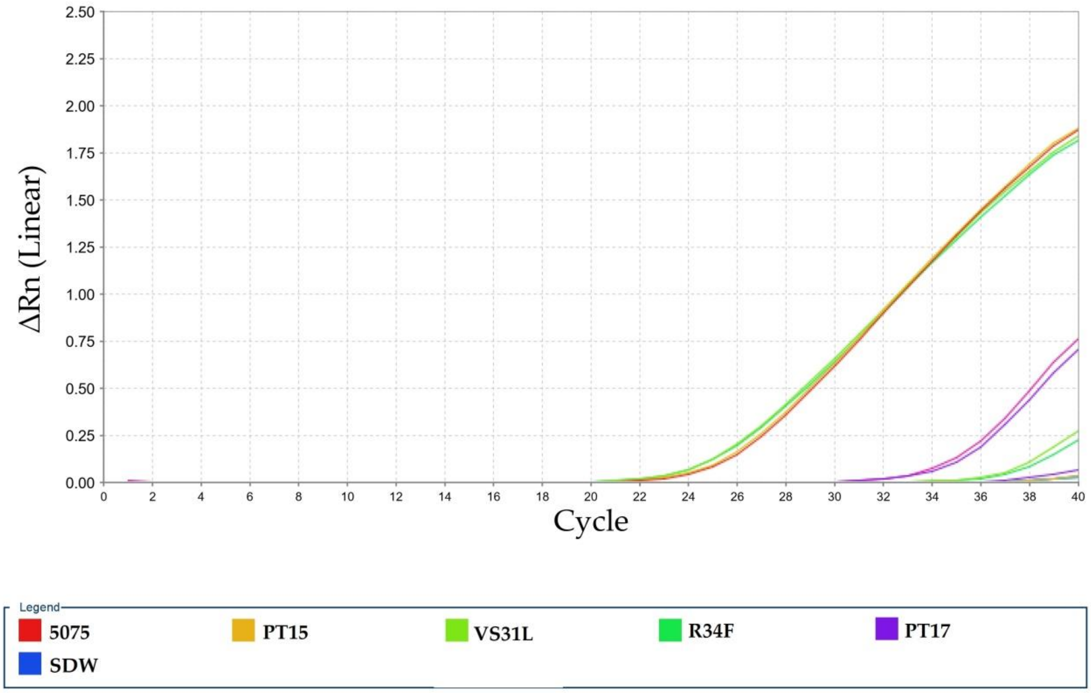
ΔRn (Linear)
2.50
2.25
2.00
1.75
1.50
1.25
1.00
0.75
0.50
0.25
0.00
0 2 4 6 8 10 12 14 16 18 20 22 24 26 28 30 32 34 36 38 40
Cycle
Legend
5075
PT15
VS31L
R34F
PT17
SDW

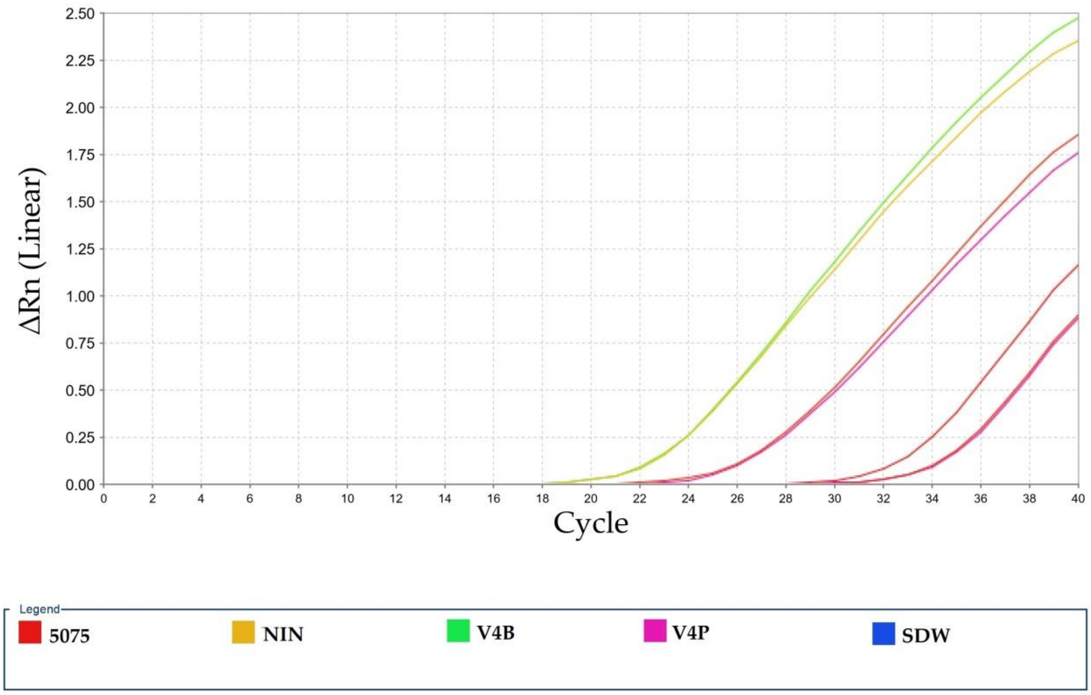

Figure S1. Amplification plots of positive Real-time PCR isolates of *Pseudomonas savastanoi* pv. *savastanoi* isolated from olive knots collected in Croatia and Portugal. X axis represents number of PCR amplification cycles, and y axis the fluorescence emission intensity of the reporter dye (VIC) divided by the fluorescence emission intensity of the passive reference dye (ROX). Endogenous control was *iaaL,* while sterile distiled water served as negative control (SDW). Positive isolates are represented with curves coloured for each isolate listed in figure legends. Codes of isolates are compliant with those listed in Table 2 in manuscript.
