# Supplementary material for: Identification and Characterisation of Pseudomonas savastanoi pv. savastanoi as the Causal Agent of Olive Knot Disease in Croatian, Slovenian and Portuguese Olive (Olea europaea L.) Orchards
Source: Plants (Basel). 2023 Jan 9;12(2):307. doi: 10.3390/plants12020307 (PMC9865541; doi:10.3390/plants12020307)
Supplement: Supplementary file 1 [file plants-12-00307-s001.zip › Figure S2. Gel electrophoresis of End Point PCR identified Pss isolates from Slovenia.pdf]

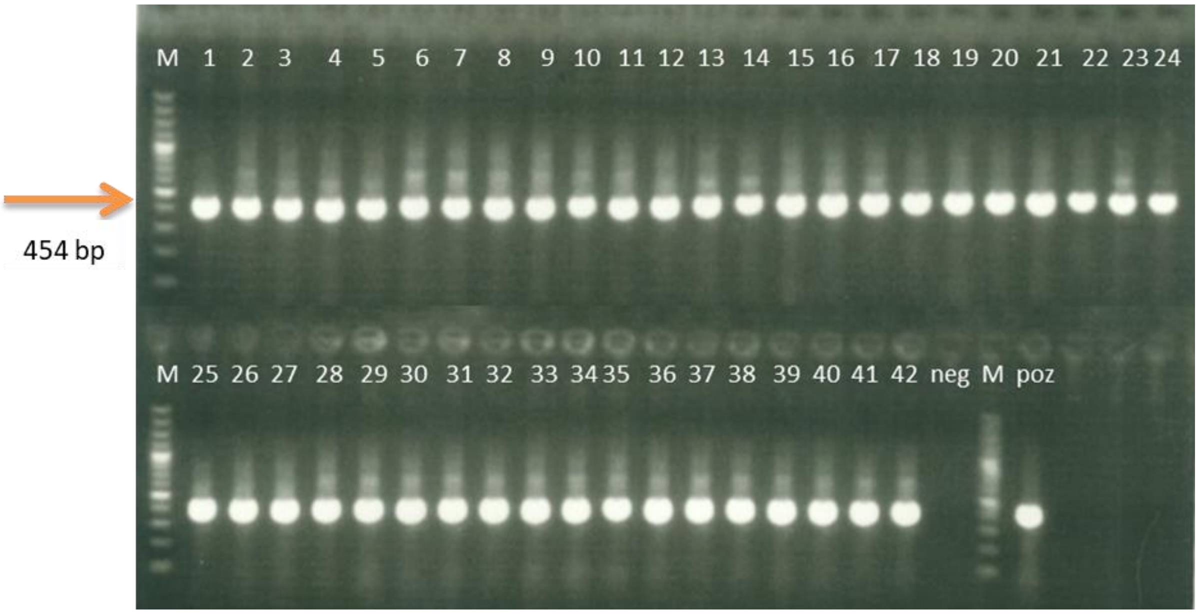

Figure S2. Agarose gel electrophoresis of the 454 bp PCR products obtained with primers IAAL-F and IAAL-R on thermally-lysed DNA (2 μL/reaction) from 42 bacterial isolates from olive knots collected in Slovenia (lanes 1-42). Lane 1, L2-4 strain; Lane 2, L2-5 strain; Lane 3, L6-2 strain; Lane 4, L6-3 strain; Lane 5, L6-4 strain; Lane 6, F3-2 strain; Lane 7, F3-3 strain; Lane 8, F3-4 strain; Lane 9, F3-5 strain; Lane 10, F3-1 strain; Lane 11, L1-1 strain; Lane 12, L6-5 strain; Lane 13, L5-3 strain; Lane 14, L5-1 strain; Lane 15, L5-5 strain; Lane 16, L5-4 strain; Lane 17, L5-2 strain; Lane 18, F4-2 strain; Lane 19, F4-1 strain; Lane 20, F4-4 strain; Lane 21, F4-3 strain; Lane 22, F4-5 strain; Lane 23, L4-1 strain; Lane 24, L4-2 strain; Lane 25, L4-4 strain; Lane 26, L4-4 strain; Lane 27, L6-1 strain; Lane 28, F2-2 strain; Lane 29, F2-4 strain; Lane 30, F2-5 strain; Lane 31, F2-3 strain; Lane 32, F2-1 strain; Lane 33, At1-2 strain; Lane 34, At1-5 strain; Lane 35, At1-1 strain; Lane 36, At1-4 strain; Lane 37, At1-3 strain; Lane 38, At2-6 strain; Lane 39, At2-1 strain; Lane 40, At2-3 strain; Lane 41, At2-4 strain; Lane 42, At2-5 strain; **M**, 100 bp DNA Ladder, ThermoFisher, USA; **poz**, positive control with the *P. savastanoi* pv. *savastanoi* reference strain CFBP5075 DNA as template; **neg**, negative control with SDW as template.
