# Supplementary material for: Identification and Characterisation of Pseudomonas savastanoi pv. savastanoi as the Causal Agent of Olive Knot Disease in Croatian, Slovenian and Portuguese Olive (Olea europaea L.) Orchards
Source: Plants (Basel). 2023 Jan 9;12(2):307. doi: 10.3390/plants12020307 (PMC9865541; doi:10.3390/plants12020307)
Supplement: Supplementary file 1 [file plants-12-00307-s001.zip › Figure S3. Bichemical and physiological characteristics of Pss isolates.pdf]

Characteristic		Percentage of *P. savastanoi* pv. *savastanoi* isolates (%)		
		**Croatian isolates**	**Slovenian isolates**	**Portuguese isolates**
**Levan production***				
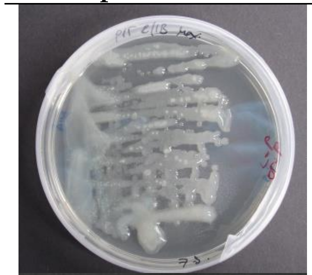	**P**	0	0	0
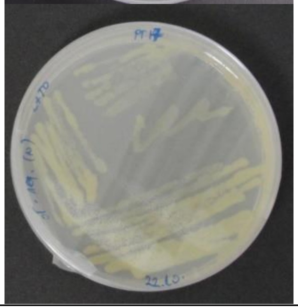	**N**	100	100	100
**Oxidase test****				
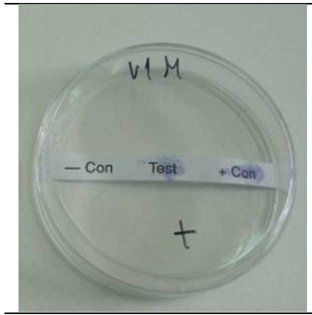	**P**	6.25 (P44F, B45C-PR)	10.00 (L1-1, F4-1, F4-4)	0
	**DP**	12.50 (17L, VS28L, VS28N, VS29L)	76.67	0
	**N**	80.45	13.33 (L6-2, L5-1, F4-2, At2-4)	100
**Pectolytic activity*****				

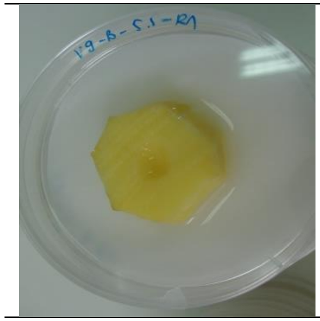	**P**	0	0	0
	**N**	100	100	100
**Arginine dihydrolase activity****				
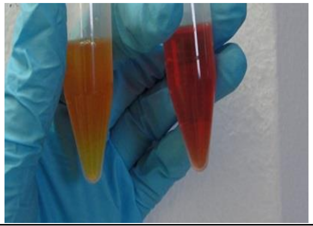	**P**	0	0	0
	**N**	100	100	100
**Tobacco hypersensitivity reaction*****				
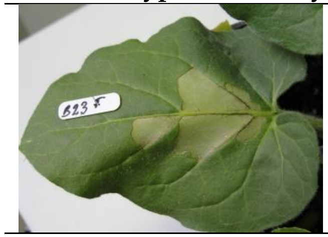	**P**	93.75	100	100
	**N**	6.25 (V4B, V4P)	0	0
**Gram reaction******				
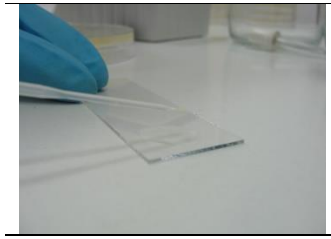	**P**	100	100	100
	**N**	0	0	0

**Fluorescence on KB medium under UV light*******				
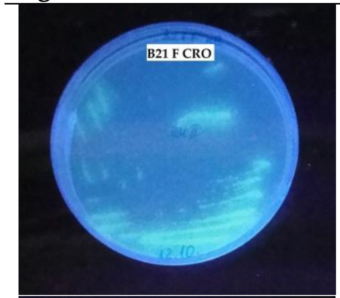	**P**	28.10 (V4P, R12L, P15TR, P15N, B19F, B19P, B21F, 16L, B45C-PR)	100	100
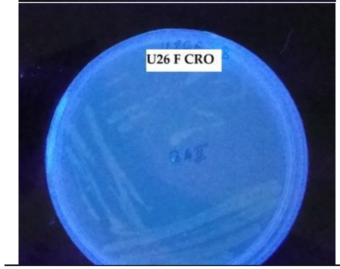	**N**	71.90	0	0

Figure S3. Biochemical and physiological characterization of molecularly identified *Pss* isolates from Croatian, Slovenian and Portuguese olive orchards based on determinative scheme for *Pseudomonas* species.

P – Positive; N – Negative; DP – Delayed positive

*Levan production – first picture represents unknown bacterium isolated from sample material P15R/IB which resulted positive for this characteristic, while picture below represents confirmed *Pss* isolate PT17 (Portugal) that resulted as negative in production of levan when grown on 5% sucrose nutrient agarised medium.

**Oxidase activity – picture represents oxidase positive bacterial sample using commercial oxidase strips.

***Pectolytic activity – picture represents absence of potato soft root when inoculated with bacterium *Pss* (isolate V9 B - Croatia).

****Arginine dihydrolase activity – left tube on the picture represents negative results for this trait and right tube positive results after incubation period of four days in Thornley's medium 2A.

*****Tobacco hypersensitivity reaction – pictures represent the collapse of tobacco leaf tissue after 24 h when *Pss* isolate B24 F is inoculated.

******Gram reaction – picture represents determination of Gram reaction using 3% potassium hydroxide on 24 h old bacterial cultures.

*******Fluorescence under UV light – Upper picture represents *Pss* isolate B21 F positive for production of fluorescent pigment under UV light (366 nm wavelength), while bottom picture represents *Pss* isolate U26 F with absence of fluorescent pigment production *in vitro*.
