# Supplementary material for: Identification and Characterisation of Pseudomonas savastanoi pv. savastanoi as the Causal Agent of Olive Knot Disease in Croatian, Slovenian and Portuguese Olive (Olea europaea L.) Orchards
Source: Plants (Basel). 2023 Jan 9;12(2):307. doi: 10.3390/plants12020307 (PMC9865541; doi:10.3390/plants12020307)
Supplement: Supplementary file 1 [file plants-12-00307-s001.zip › Figure S4. Colony morphology of tested Pss isolates.pdf]

Isolate	Morphological trait					
**Code**	**Diameter (mm)**	**Shape**	**Margins**	**Colony center**	**Pigment**	**Colony figure**
**CFBP5075 Reference strain**	1-2	Round and oval	Smooth	Dark	White-grey	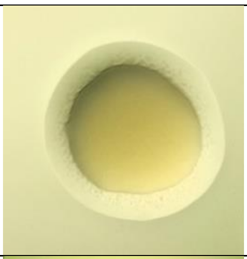
**PT3 Negative isolate**	1-3	Round	Smooth	Light	Intensive yellow	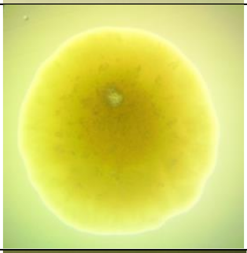
**V4B**	2	Round	Smooth	Dark	Yellow	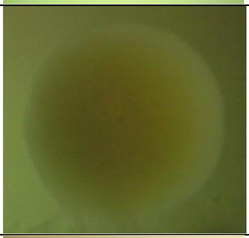
**V4P**	2-3	Round and oval	Smooth and corrugated	Dark	White-gray	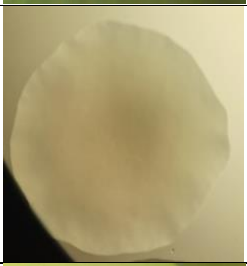
**V7K**	2-3	Round and oval	Smooth	Dark	Yellow	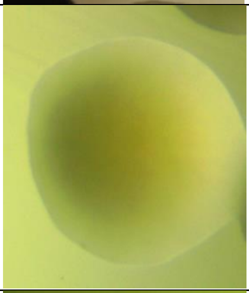
**V9B**	2-3	Round	Smooth	Dark	Yellow	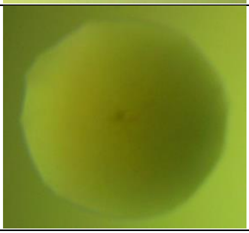

**R12L**	2-3	Round and oval	Smooth	Dark	White-grey	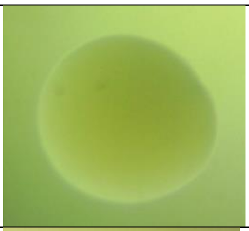
**P15TR**	1-3	Round	Smooth	Dark	White-grey	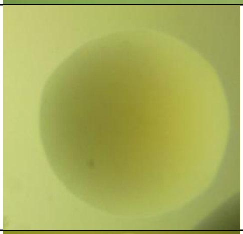
**P15N**	2-3	Round	Smooth	Dark	White-grey	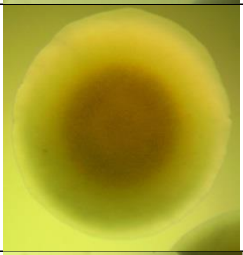
**B17L**	1-2	Round and oval	Smooth	Dark	Yellow	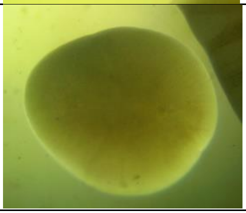
**B19F**	3	Round	Smooth	Dark	White-grey	-
**B19L**	2	Round	Smooth	Dark	Yellow	-
**B19P**	2-3	Round and oval	Smooth	Light	White-grey	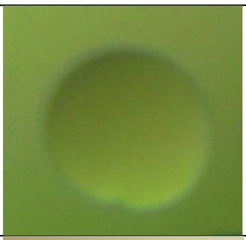
**B21L**	1-2	Round	Smooth	Light	Yellow	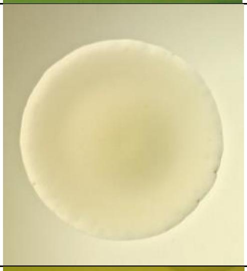
**B21F**	1.-3	Round and oval	Smooth	Dark	White-grey	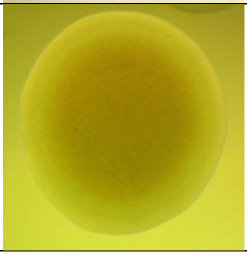

**B23F**	1-2	Round	Smooth	Dark	White-grey	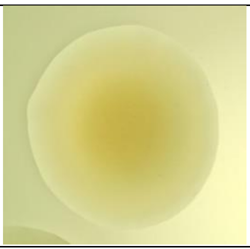
**16L**	2-3	Round	Smooth and corrugated	Dark	White-grey	-
**17L**	1-2	Round	Smooth	Dark	White-grey	-
**NIN**	2	Round	Smooth	Dark	White-grey	-
**U26F**	2	Round	Smooth and corrugated	Dark	Yellow	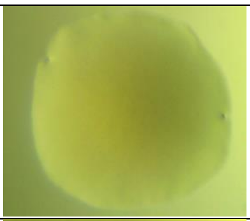
**U27F**	2-3	Round and oval	Smooth	Dark	Yellow	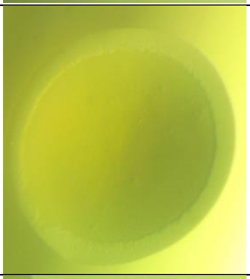
**VS28N**	2-3	Round and oval	Smooth and corrugated	Dark	Yellow	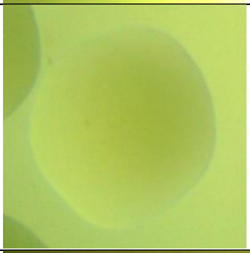
**VS28L**	1-2	Round	Smooth and corrugated	Dark	Yellow	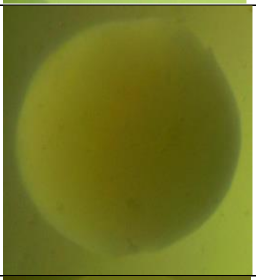
**VS29L**	1-2	Round	Smooth	Dark	White-grey	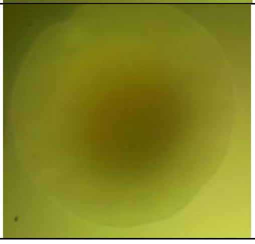

**VS31L**	1-2	Round and oval	Smooth and corrugated	Dark	White-grey	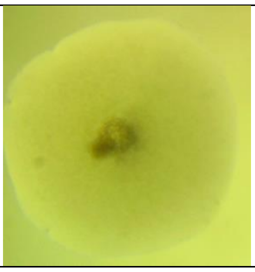
**R34F**	2-3	Round and oval	Smooth	Light	White-grey	-
**BR37L**	2	Round	Smooth and corrugated	Light	Yellow	-
**N40M**	2-3	Round and oval	Smooth	Light	Yellow	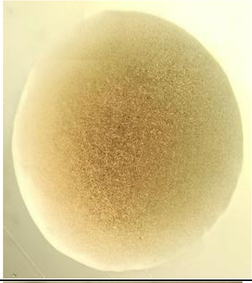
**N41F**	2-3	Round and oval	Smooth	Dark	Yellow	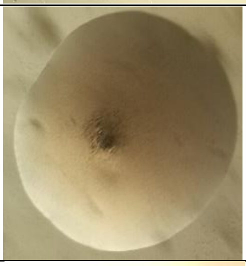
**N42F**	2-3	Round and ova	Smooth	Light	Yellow	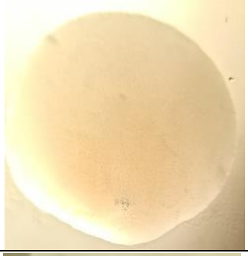
**N43L**	2	Round	Smooth	Dark	White-grey	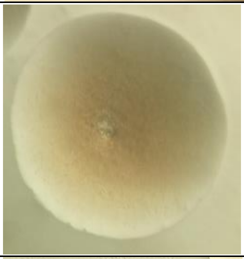
**N43P**	2-3	Round and oval	Smooth	Dark	White-grey	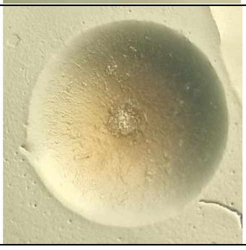
**P44F**	2-3	Round	Smooth	Dark	White-grey	-
**B45C-PR**	2-3	Round	Smooth	Dark	White-grey	-

**PT15**	1-2	Round	Smooth	Light	Yellow	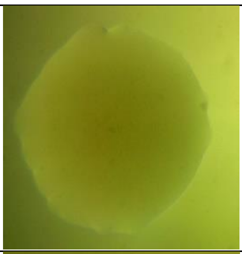
**PT17**	1-2	Round	Smooth	Dark	Yellow	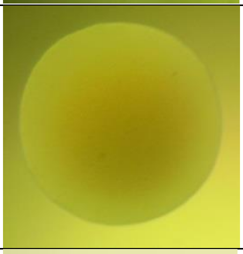
**Slovenian isolates**	0.5-1	Round	Smooth	Light	White-grey	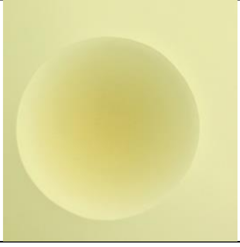

Figure S4. Colony morphology of bacterial colonies of molecularly identified *Pss* compared to *Pss* reference strain CFBP5075 (Italy) and unknown bacterial isolate PT3 after 48 h of growth on agarised King's B growth medium.
