# Supplementary material for: Identification and Characterisation of Pseudomonas savastanoi pv. savastanoi as the Causal Agent of Olive Knot Disease in Croatian, Slovenian and Portuguese Olive (Olea europaea L.) Orchards
Source: Plants (Basel). 2023 Jan 9;12(2):307. doi: 10.3390/plants12020307 (PMC9865541; doi:10.3390/plants12020307)
Supplement: Supplementary file 1 [file plants-12-00307-s001.zip › Supplementary S1. Slovenian PCR positive Pss isolates confirmed using mass spectrometry.pdf]

Bruker Daltonik MALDI Biotyper Classification Results

Project Info:

Project Name:	**20211119d KIS 11-21-60x**
Project Description:	
Project Owner:	admin@FLEX-PC
Project Creation Date/Time:	2021-11-19T14:07:42.862
Project Analyte Count:	60
Project Type:	Development
Validation:	not present
Validation Position:	

Result Overview

Analyte Name	Analyte ID	Organism (best match)	Score Value	Organism (second best match)	Score Value
A1 (+++) (C)	1	Pseudomonas savastanoi	2.309	Pseudomonas savastanoi	2.039
A2 (++) (C)	2	Pseudomonas savastanoi	2.289	Pseudomonas savastanoi	2.087
A3 (++) (C)	3	Pseudomonas savastanoi	2.257	Pseudomonas savastanoi	2.034
A4 (+++) (C)	4	Pseudomonas savastanoi	2.337	Pseudomonas savastanoi	2.075
A5 (+++) (C)	5	Pseudomonas savastanoi	2.334	Pseudomonas savastanoi	2.103
A6 (+++) (C)	6	Pseudomonas savastanoi	2.378	Pseudomonas savastanoi	2.214
A7 (+++) (C)	7	Pseudomonas savastanoi	2.316	Pseudomonas savastanoi	2.126
A8 (+++) (C)	8	Pseudomonas savastanoi	2.325	Pseudomonas savastanoi	2.184
A9 (++) (C)	9	Pseudomonas savastanoi	2.263	Pseudomonas savastanoi	2.175

A10 (++) (C)	10	Pseudomonas savastanoi	2.293	Pseudomonas savastanoi	2.1
A11 (++) (C)	11	Pseudomonas savastanoi	2.288	Pseudomonas savastanoi	2.005
A12 (+++) (C)	12	Pseudomonas savastanoi	2.37	Pseudomonas savastanoi	2.096
B1 (+++) (C)	13	Pseudomonas savastanoi	2.315	Pseudomonas savastanoi	2.122
B2 (++) (C)	14	Pseudomonas savastanoi	2.206	Pseudomonas savastanoi	2.053
B3 (++) (C)	15	Pseudomonas savastanoi	2.293	Pseudomonas savastanoi	2.23
B4 (++) (C)	16	Pseudomonas savastanoi	2.278	Pseudomonas savastanoi	2.115
B5 (+++) (C)	17	Pseudomonas savastanoi	2.335	Pseudomonas savastanoi	2.183
B6 (++) (C)	18	Pseudomonas savastanoi	2.238	Pseudomonas savastanoi	2.125
B7 (++) (C)	19	Pseudomonas savastanoi	2.294	Pseudomonas savastanoi	2.214
B8 (+++) (C)	20	Pseudomonas savastanoi	2.37	Pseudomonas savastanoi	2.228
B9 (++) (C)	21	Pseudomonas savastanoi	2.243	Pseudomonas savastanoi	2.173
B10 (++) (C)	22	Pseudomonas savastanoi	2.267	Pseudomonas savastanoi	2.162
B11 (+++) (C)	23	Pseudomonas savastanoi	2.383	Pseudomonas savastanoi	2.143
B12 (+++) (C)	24	Pseudomonas savastanoi	2.308	Pseudomonas savastanoi	2.194
C1 (+++) (C)	25	Pseudomonas savastanoi	2.314	Pseudomonas savastanoi	2.246
C2 (++) (C)	26	Pseudomonas savastanoi	2.289	Pseudomonas savastanoi	2.095
C3 (+++) (C)	27	Pseudomonas savastanoi	2.32	Pseudomonas savastanoi	2.188
C4 (++) (C)	28	Pseudomonas savastanoi	2.257	Pseudomonas savastanoi	2.151
C5 (++) (C)	29	Pseudomonas savastanoi	2.291	Pseudomonas savastanoi	2.183
C6	30	Pseudomonas savastanoi	2.297	Pseudomonas savastanoi	2.249

(++) (C)					
C7 (++) (C)	31	Pseudomonas savastanoi	2.276	Pseudomonas savastanoi	2.235
C8 (++) (C)	32	Pseudomonas savastanoi	2.297	Pseudomonas savastanoi	2.283
C9 (+++) (C)	33	Pseudomonas savastanoi	2.334	Pseudomonas savastanoi	2.238
C10 (++) (C)	34	Pseudomonas savastanoi	2.178	Pseudomonas savastanoi	2.143
C11 (++) (C)	35	Pseudomonas savastanoi	2.291	Pseudomonas savastanoi	2.167
C12 (++) (C)	36	Pseudomonas savastanoi	2.26	Pseudomonas savastanoi	2.169
D1 (-) (C)	37	**no peaks found**	< 0	**no peaks found**	< 0
D2 (++) (C)	38	Pseudomonas savastanoi	2.265	Pseudomonas savastanoi	2.183
D3 (++) (C)	39	Pseudomonas savastanoi	2.256	Pseudomonas savastanoi	2.123
D4 (++) (C)	40	Pseudomonas savastanoi	2.278	Pseudomonas savastanoi	2.219
D5 (+++) (C)	41	Pseudomonas savastanoi	2.369	Pseudomonas savastanoi	2.115
D6 (++) (C)	42	Pseudomonas savastanoi	2.283	Pseudomonas savastanoi	2.061
D7 (+++) (C)	43	Pseudomonas savastanoi	2.351	Pseudomonas savastanoi	2.183
D8 (+++) (C)	44	Pseudomonas savastanoi	2.347	Pseudomonas savastanoi	2.173
D9 (++) (C)	45	Pseudomonas savastanoi	2.228	Pseudomonas savastanoi	2.113
D10 (+++) (C)	46	Pseudomonas savastanoi	2.357	Pseudomonas savastanoi	2.168
D11 (+++) (C)	47	Pseudomonas savastanoi	2.325	Pseudomonas savastanoi	2.087
D12 (+++) (C)	48	Pseudomonas savastanoi	2.321	Pseudomonas savastanoi	2.078
E1 (++) (C)	49	Pseudomonas savastanoi	2.289	Pseudomonas savastanoi	2.092
E2 (-) (C)	50	**not reliable identification**	1.599	**not reliable identification**	1.377

E3<br>**(+++) (C)**	51	Pseudomonas savastanoi	2.361	Pseudomonas savastanoi	2.181
E4<br>**(-) (C)**	52	**not reliable identification**	1.604	**not reliable identification**	1.502
E5<br>**(-) (C)**	53	**not reliable identification**	1.688	**not reliable identification**	1.304
E6<br>**(+++) (A)**	54	Bacillus megaterium	2.391	Bacillus megaterium	2.341
E7<br>**(-) (C)**	55	**not reliable identification**	1.642	**not reliable identification**	1.253
E8<br>**(++) (C)**	56	Pseudomonas savastanoi	2.292	Pseudomonas savastanoi	2.062
E9<br>**(+++) (C)**	57	Pseudomonas savastanoi	2.304	Pseudomonas savastanoi	2.127
E10<br>**(+++) (C)**	58	Pseudomonas savastanoi	2.3	Pseudomonas savastanoi	2.141
E11<br>**(++) (C)**	59	Pseudomonas savastanoi	2.244	Pseudomonas caricapapayae	2.02
E12<br>**(+++) (C)**	60	Pseudomonas savastanoi	2.316	Pseudomonas savastanoi	2.136

Matching Hints

Matched Pattern	Comment
Bacillus cohnii DSM 6307T DSM	The quality of spectra (score) depends on the degree of sporulation: Use fresh material.
Bacillus megaterium DSM 1668 DSM	The quality of spectra (score) depends on the degree of sporulation: Use fresh material.
Bacillus megaterium DSM 2894 DSM	The quality of spectra (score) depends on the degree of sporulation: Use fresh material.
Bacillus megaterium DSM 32T DSM	The quality of spectra (score) depends on the degree of sporulation: Use fresh material.
Bacillus sonorensis DSM 13779T DSM	is a member of Bacillus subtilis group. The quality of spectra (score) depends on the degree of sporulation: Use fresh material.
Citrobacter freundii 22054_1 CHB	Species of this genus have very similar patterns: Therefore distinguishing their species is difficult.
Lactobacillus amylovorus DSM 20531T DSM	Species acidophilus / amylovorus / kitasatonis of the genus Lactobacillus have very similar patterns: Therefore distinguishing their species is difficult.
Proteus vulgaris (PX) 22086129 MLD	Species hauseri / penneri / vulgaris of the genus Proteus have very similar patterns: Therefore distinguishing their species is difficult.
Pseudomonas congelans DSM 14939T HAM	is a member of Pseudomonas fluorescens group

Meaning of Score Values

Range	Description	Symbols	Color
**2.300 ... 3.000**	**highly probable species identification**	**(+++)**	green
**2.000 ... 2.299**	**secure genus identification, probable species identification**	**(++)**	green
**1.700 ... 1.999**	**probable genus identification**	**(+)**	yellow
**0.000 ... 1.699**	**not reliable identification**	**(-)**	red

Meaning of Consistency Categories (A - C)

Category	Description
**A**	**Species Consistency:** The best match was classified as 'green' (see above). Further 'green' matches are of the same species as the first one. Further 'yellow' matches are at least of the same genus as the first one.
**B**	**Genus Consistency:** The best match was classified as 'green' or 'yellow' (see above). Further 'green' or 'yellow' matches have at least the same genus as the first one. The conditions of species consistency are not fulfilled.
**C**	**No Consistency:** Neither species nor genus consistency (Please check for synonyms of names or microbial mixture).

Analyte1

BRUKER

Analyte Name:	A1
Analyte Description:	
Analyte ID:	1
Analyte Creation Date/Time:	2021-11-19T14:09:10.682
Applied MSP Library(ies):	
Applied Taxonomy Tree:	Projects, Bruker Taxonomy

Rank (Quality)	Matched Pattern	Score Value	NCBI Identifier
1 (+++)	Pseudomonas savastanoi ssp savastanoi LMG 2209T HAM	2.309	29438

2<br>(++)	Pseudomonas savastanoi ssp savastanoi LMG 5011 HAM	2.039	29438
3<br>(+)	Pseudomonas syringae ssp syringae DSM 6693 HAM	1.898	317
4<br>(+)	2501	1.793	451320
5<br>(+)	Pseudomonas agarici DSM 11810T HAM	1.738	46677
6<br>(+)	Pseudomonas congelans DSM 14939T HAM	1.71	200452
7<br>(-)	Pseudomonas caricapapayae LMG 2152T HAM	1.613	46678
8<br>(-)	Pseudomonas avellanae DSM 11809T DSM	1.607	46257
9<br>(-)	2502	1.567	451320
10<br>(-)	Pseudomonas chlororaphis ssp aurantiaca CIP 106718T HAM	1.519	333

Analyte2

Analyte Name:	A2
Analyte Description:	
Analyte ID:	2
Analyte Creation Date/Time:	2021-11-19T14:09:09.859
Applied MSP Library(ies):	
Applied Taxonomy Tree:	Projects, Bruker Taxonomy

Rank (Quality)	Matched Pattern	Score Value	NCBI Identifier
1 (++)	Pseudomonas savastanoi ssp savastanoi LMG 2209T HAM	2.289	29438
2 (++)	Pseudomonas savastanoi ssp savastanoi LMG 5011 HAM	2.087	29438
3 (+)	Pseudomonas agarici DSM 11810T HAM	1.768	46677
4 (+)	2501	1.753	451320
5 (+)	Pseudomonas congelans DSM 14939T HAM	1.737	200452
6 (+)	Pseudomonas syringae ssp syringae DSM 6693 HAM	1.73	317
7 (-)	2502	1.674	451320
8 (-)	Pseudomonas caricapapayae LMG 2152T HAM	1.505	46678
9 (-)	Pseudomonas cichorii DSM 50259T HAM	1.499	36746
10 (-)	Pseudomonas chlororaphis ssp chlororaphis DSM 50083T HAM	1.48	333

Analyte3

Analyte Name:	A3
Analyte Description:	
Analyte ID:	3
Analyte Creation Date/Time:	2021-11-19T14:09:10.884
Applied MSP Library(ies):	
Applied Taxonomy Tree:	Projects, Bruker Taxonomy

Rank (Quality)	Matched Pattern	Score Value	NCBI Identifier
1 (++)	Pseudomonas savastanoi ssp savastanoi LMG 2209T HAM	2.257	29438
2 (++)	Pseudomonas savastanoi ssp savastanoi LMG 5011 HAM	2.034	29438
3 (+)	Pseudomonas caricapapayae LMG 2152T HAM	1.902	46678
4 (+)	Pseudomonas avellanae DSM 11809T DSM	1.883	46257
5 (+)	2501	1.881	451320
6 (+)	Pseudomonas syringae ssp syringae DSM 6693 HAM	1.855	317
7 (+)	Pseudomonas congelans DSM 14939T HAM	1.843	200452
8 (+)	2502	1.837	451320
9 (+)	Pseudomonas agarici DSM 11810T HAM	1.765	46677
10 (-)	Pseudomonas chlororaphis ssp aurantiaca CIP 106718T HAM	1.68	333

Analyte4

Analyte Name:	A4
Analyte Description:	
Analyte ID:	4
Analyte Creation Date/Time:	2021-11-19T14:09:10.422
Applied MSP Library(ies):	
Applied Taxonomy Tree:	Projects, Bruker Taxonomy

Rank (Quality)	Matched Pattern	Score Value	NCBI Identifier
1 (+++)	Pseudomonas savastanoi ssp savastanoi LMG 2209T HAM	2.337	29438
2 (++)	Pseudomonas savastanoi ssp savastanoi LMG 5011 HAM	2.075	29438
3 (+)	Pseudomonas syringae ssp syringae DSM 6693 HAM	1.925	317
4 (+)	Pseudomonas caricapapayae LMG 2152T HAM	1.918	46678
5 (+)	Pseudomonas agarici DSM 11810T HAM	1.81	46677
6 (+)	2501	1.792	451320
7 (+)	2502	1.785	451320
8 (+)	Pseudomonas congelans DSM 14939T HAM	1.782	200452
9 (+)	Pseudomonas avellanae DSM 11809T DSM	1.731	46257
10 (-)	Pseudomonas cichorii DSM 50259T HAM	1.663	36746

Analyte5

BRUKER

Analyte Name:	A5
Analyte Description:	
Analyte ID:	5
Analyte Creation Date/Time:	2021-11-19T14:09:10.247
Applied MSP Library(ies):	
Applied Taxonomy Tree:	Projects, Bruker Taxonomy

Rank (Quality)	Matched Pattern	Score Value	NCBI Identifier
1 (+++)	Pseudomonas savastanoi ssp savastanoi LMG 2209T HAM	2.334	29438
2 (++)	Pseudomonas savastanoi ssp savastanoi LMG 5011 HAM	2.103	29438
3 (+)	Pseudomonas caricapapayae LMG 2152T HAM	1.967	46678
4 (+)	2501	1.891	451320
5 (+)	Pseudomonas syringae ssp syringae DSM 6693 HAM	1.859	317
6 (+)	2502	1.851	451320
7 (+)	Pseudomonas congelans DSM 14939T HAM	1.832	200452
8 (+)	Pseudomonas agarici DSM 11810T HAM	1.771	46677
9 (+)	Pseudomonas avellanae DSM 11809T DSM	1.764	46257
10 (-)	Pseudomonas cichorii DSM 50259T HAM	1.68	36746

Analyte6

Analyte Name:	A6
Analyte Description:	
Analyte ID:	6
Analyte Creation Date/Time:	2021-11-19T14:09:09.745
Applied MSP Library(ies):	
Applied Taxonomy Tree:	Projects, Bruker Taxonomy

Rank (Quality)	Matched Pattern	Score Value	NCBI Identifier
1 (+++)	Pseudomonas savastanoi ssp savastanoi LMG 2209T HAM	2.378	29438
2 (++)	Pseudomonas savastanoi ssp savastanoi LMG 5011 HAM	2.214	29438
3 (++)	Pseudomonas caricapapayae LMG 2152T HAM	2.012	46678
4 (+)	Pseudomonas syringae ssp syringae DSM 6693 HAM	1.879	317
5 (+)	2501	1.862	451320
6 (+)	Pseudomonas congelans DSM 14939T HAM	1.841	200452
7 (+)	Pseudomonas chlororaphis ssp aurantiaca CIP 106718T HAM	1.769	333
8 (+)	Pseudomonas avellanae DSM 11809T DSM	1.748	46257
9 (+)	Pseudomonas cichorii DSM 50259T HAM	1.708	36746
10 (-)	Pseudomonas chlororaphis ssp chlororaphis DSM 50083T HAM	1.692	333

Analyte7

Analyte Name:	A7
Analyte Description:	
Analyte ID:	7
Analyte Creation Date/Time:	2021-11-19T14:09:09.694
Applied MSP Library(ies):	
Applied Taxonomy Tree:	Bruker Taxonomy, Projects

Rank (Quality)	Matched Pattern	Score Value	NCBI Identifier
1 (+++)	Pseudomonas savastanoi ssp savastanoi LMG 2209T HAM	2.316	29438
2 (++)	Pseudomonas savastanoi ssp savastanoi LMG 5011 HAM	2.126	29438
3 (+)	Pseudomonas caricapapayae LMG 2152T HAM	1.911	46678
4 (+)	Pseudomonas syringae ssp syringae DSM 6693 HAM	1.873	317
5 (+)	2501	1.86	451320
6 (+)	Pseudomonas agarici DSM 11810T HAM	1.845	46677
7 (+)	Pseudomonas congelans DSM 14939T HAM	1.835	200452
8 (+)	2502	1.763	451320
9 (-)	Pseudomonas cichorii DSM 50259T HAM	1.693	36746
10 (-)	Pseudomonas chlororaphis ssp aurantiaca CIP 106718T HAM	1.644	333

Analyte8

Analyte Name:	A8
Analyte Description:	
Analyte ID:	8
Analyte Creation Date/Time:	2021-11-19T14:09:10.667
Applied MSP Library(ies):	
Applied Taxonomy Tree:	Projects, Bruker Taxonomy

Rank (Quality)	Matched Pattern	Score Value	NCBI Identifier
1 (+++)	Pseudomonas savastanoi ssp savastanoi LMG 2209T HAM	2.325	29438
2 (++)	Pseudomonas savastanoi ssp savastanoi LMG 5011 HAM	2.184	29438
3 (+)	Pseudomonas caricapapayae LMG 2152T HAM	1.96	46678
4 (+)	Pseudomonas congelans DSM 14939T HAM	1.895	200452
5 (+)	Pseudomonas syringae ssp syringae DSM 6693 HAM	1.893	317
6 (+)	2501	1.851	451320
7 (+)	2502	1.778	451320
8 (+)	Pseudomonas cichorii DSM 50259T HAM	1.725	36746
9 (-)	Pseudomonas avellanae DSM 11809T DSM	1.692	46257
10 (-)	Pseudomonas viridiflava DSM 11124T HAM	1.661	33069

Analyte9

Analyte Name:	A9
Analyte Description:	
Analyte ID:	9
Analyte Creation Date/Time:	2021-11-19T14:09:09.828
Applied MSP Library(ies):	
Applied Taxonomy Tree:	Projects, Bruker Taxonomy

Rank (Quality)	Matched Pattern	Score Value	NCBI Identifier
1 (++)	Pseudomonas savastanoi ssp savastanoi LMG 2209T HAM	2.263	29438
2 (++)	Pseudomonas savastanoi ssp savastanoi LMG 5011 HAM	2.175	29438
3 (+)	Pseudomonas caricapapayae LMG 2152T HAM	1.932	46678
4 (+)	2501	1.861	451320
5 (+)	Pseudomonas agarici DSM 11810T HAM	1.728	46677
6 (-)	Pseudomonas chlororaphis ssp aurantiaca CIP 106718T HAM	1.678	333
7 (-)	Pseudomonas avellanae DSM 11809T DSM	1.67	46257
8 (-)	Pseudomonas congelans DSM 14939T HAM	1.661	200452
9 (-)	Pseudomonas syringae ssp syringae DSM 6693 HAM	1.638	317
10 (-)	2502	1.634	451320

Analyte10

Analyte Name: A10
Analyte Description:
Analyte ID: 10
Analyte Creation Date/Time: 2021-11-19T14:09:10.917
Applied MSP Library(ies):
Applied Taxonomy Tree: Projects, Bruker Taxonomy

Rank (Quality)	Matched Pattern	Score Value	NCBI Identifier
1 (++)	Pseudomonas savastanoi ssp savastanoi LMG 2209T HAM	2.293	29438
2 (++)	Pseudomonas savastanoi ssp savastanoi LMG 5011 HAM	2.1	29438
3 (+)	Pseudomonas caricapapayae LMG 2152T HAM	1.867	46678
4 (+)	2501	1.85	451320
5 (+)	Pseudomonas congelans DSM 14939T HAM	1.84	200452
6 (+)	Pseudomonas agarici DSM 11810T HAM	1.801	46677
7 (+)	Pseudomonas syringae ssp syringae DSM 6693 HAM	1.791	317
8 (+)	2502	1.769	451320
9 (-)	Pseudomonas avellanae DSM 11809T DSM	1.692	46257
10 (-)	Pseudomonas cichorii DSM 50259T HAM	1.536	36746

Analyte11

Analyte Name:	A11
Analyte Description:	
Analyte ID:	11
Analyte Creation Date/Time:	2021-11-19T14:09:10.364
Applied MSP Library(ies):	
Applied Taxonomy Tree:	Bruker Taxonomy, Projects

Rank (Quality)	Matched Pattern	Score Value	NCBI Identifier
1 (++)	Pseudomonas savastanoi ssp savastanoi LMG 2209T HAM	2.288	29438
2 (++)	Pseudomonas savastanoi ssp savastanoi LMG 5011 HAM	2.005	29438
3 (+)	Pseudomonas syringae ssp syringae DSM 6693 HAM	1.894	317
4 (+)	Pseudomonas avellanae DSM 11809T DSM	1.812	46257
5 (+)	Pseudomonas agarici DSM 11810T HAM	1.766	46677
6 (+)	2501	1.705	451320
7 (-)	Pseudomonas congelans DSM 14939T HAM	1.685	200452
8 (-)	Pseudomonas chlororaphis ssp aurantiaca CIP 106718T HAM	1.64	333
9 (-)	Pseudomonas cichorii DSM 50259T HAM	1.584	36746
10 (-)	2502	1.549	451320

Analyte12

Analyte Name:	A12
Analyte Description:	
Analyte ID:	12
Analyte Creation Date/Time:	2021-11-19T14:09:10.532
Applied MSP Library(ies):	
Applied Taxonomy Tree:	Projects, Bruker Taxonomy

Rank (Quality)	Matched Pattern	Score Value	NCBI Identifier
1 (+++)	Pseudomonas savastanoi ssp savastanoi LMG 2209T HAM	2.37	29438
2 (++)	Pseudomonas savastanoi ssp savastanoi LMG 5011 HAM	2.096	29438
3 (+)	Pseudomonas syringae ssp syringae DSM 6693 HAM	1.959	317
4 (+)	Pseudomonas agarici DSM 11810T HAM	1.839	46677
5 (+)	2501	1.812	451320
6 (+)	Pseudomonas congelans DSM 14939T HAM	1.806	200452
7 (+)	Pseudomonas syringae ssp syringae LMG 1247T HAM	1.782	317
8 (+)	Pseudomonas avellanae DSM 11809T DSM	1.716	46257
9 (-)	2502	1.698	451320
10 (-)	Pseudomonas caricapapayae LMG 2152T HAM	1.618	46678

Analyte13

Analyte Name:	B1
Analyte Description:	
Analyte ID:	13
Analyte Creation Date/Time:	2021-11-19T14:09:09.761
Applied MSP Library(ies):	
Applied Taxonomy Tree:	Projects, Bruker Taxonomy

Rank (Quality)	Matched Pattern	Score Value	NCBI Identifier
1 (+++)	Pseudomonas savastanoi ssp savastanoi LMG 2209T HAM	2.315	29438
2 (++)	Pseudomonas savastanoi ssp savastanoi LMG 5011 HAM	2.122	29438
3 (+)	Pseudomonas syringae ssp syringae DSM 6693 HAM	1.91	317
4 (+)	Pseudomonas caricapapayae LMG 2152T HAM	1.882	46678
5 (+)	2501	1.876	451320
6 (+)	2502	1.748	451320
7 (+)	Pseudomonas agarici DSM 11810T HAM	1.743	46677
8 (+)	Pseudomonas congelans DSM 14939T HAM	1.738	200452
9 (+)	Pseudomonas avellanae DSM 11809T DSM	1.734	46257
10 (-)	Pseudomonas syringae ssp syringae LMG 1247T HAM	1.66	317

Analyte14

BRUKER

Analyte Name:	B2
Analyte Description:	
Analyte ID:	14
Analyte Creation Date/Time:	2021-11-19T14:09:09.945
Applied MSP Library(ies):	
Applied Taxonomy Tree:	Projects, Bruker Taxonomy

Rank (Quality)	Matched Pattern	Score Value	NCBI Identifier
1 (++)	Pseudomonas savastanoi ssp savastanoi LMG 2209T HAM	2.206	29438
2 (++)	Pseudomonas savastanoi ssp savastanoi LMG 5011 HAM	2.053	29438
3 (+)	2501	1.919	451320
4 (+)	Pseudomonas caricapapayae LMG 2152T HAM	1.89	46678
5 (+)	Pseudomonas syringae ssp syringae DSM 6693 HAM	1.773	317
6 (+)	Pseudomonas agarici DSM 11810T HAM	1.717	46677
7 (+)	Pseudomonas avellanae DSM 11809T DSM	1.7	46257
8 (-)	Pseudomonas kilonensis DSM 13647T HAM	1.657	132476
9 (-)	Pseudomonas thivervalensis DSM 13194T HAM	1.647	86265
10 (-)	Pseudomonas chlororaphis ssp chlororaphis DSM 50083T HAM	1.641	333

Analyte15

Analyte Name:	B3
Analyte Description:	
Analyte ID:	15
Analyte Creation Date/Time:	2021-11-19T14:09:09.619
Applied MSP Library(ies):	
Applied Taxonomy Tree:	Projects, Bruker Taxonomy

Rank (Quality)	Matched Pattern	Score Value	NCBI Identifier
1 (++)	Pseudomonas savastanoi ssp savastanoi LMG 2209T HAM	2.293	29438
2 (++)	Pseudomonas savastanoi ssp savastanoi LMG 5011 HAM	2.23	29438
3 (++)	Pseudomonas caricapapayae LMG 2152T HAM	2.06	46678
4 (+)	Pseudomonas syringae ssp syringae DSM 6693 HAM	1.92	317
5 (+)	2501	1.901	451320
6 (+)	Pseudomonas congelans DSM 14939T HAM	1.866	200452
7 (+)	Pseudomonas cichorii DSM 50259T HAM	1.777	36746
8 (+)	2502	1.713	451320
9 (-)	Pseudomonas agarici DSM 11810T HAM	1.64	46677
10 (-)	Pseudomonas avellanae DSM 11809T DSM	1.64	46257

Analyte16

Analyte Name:	B4
Analyte Description:	
Analyte ID:	16
Analyte Creation Date/Time:	2021-11-19T14:09:09.970
Applied MSP Library(ies):	
Applied Taxonomy Tree:	Bruker Taxonomy, Projects

Rank (Quality)	Matched Pattern	Score Value	NCBI Identifier
1 (++)	Pseudomonas savastanoi ssp savastanoi LMG 2209T HAM	2.278	29438
2 (++)	Pseudomonas savastanoi ssp savastanoi LMG 5011 HAM	2.115	29438
3 (+)	2501	1.916	451320
4 (+)	Pseudomonas caricapapayae LMG 2152T HAM	1.902	46678
5 (+)	Pseudomonas syringae ssp syringae DSM 6693 HAM	1.889	317
6 (+)	2502	1.884	451320
7 (+)	Pseudomonas avellanae DSM 11809T DSM	1.842	46257
8 (+)	Pseudomonas agarici DSM 11810T HAM	1.823	46677
9 (+)	Pseudomonas congelans DSM 14939T HAM	1.751	200452
10 (-)	Pseudomonas cichorii DSM 50259T HAM	1.681	36746

Analyte17

Analyte Name:	B5
Analyte Description:	
Analyte ID:	17
Analyte Creation Date/Time:	2021-11-19T14:09:11.010
Applied MSP Library(ies):	
Applied Taxonomy Tree:	Bruker Taxonomy, Projects

Rank (Quality)	Matched Pattern	Score Value	NCBI Identifier
1 (+++)	Pseudomonas savastanoi ssp savastanoi LMG 2209T HAM	2.335	29438
2 (++)	Pseudomonas savastanoi ssp savastanoi LMG 5011 HAM	2.183	29438
3 (++)	2501	2.069	451320
4 (++)	Pseudomonas caricapapayae LMG 2152T HAM	2.034	46678
5 (+)	2502	1.98	451320
6 (+)	Pseudomonas syringae ssp syringae DSM 6693 HAM	1.925	317
7 (+)	Pseudomonas congelans DSM 14939T HAM	1.912	200452
8 (+)	Pseudomonas avellanae DSM 11809T DSM	1.876	46257
9 (+)	Pseudomonas agarici DSM 11810T HAM	1.805	46677
10 (+)	Pseudomonas syringae ssp syringae LMG 1247T HAM	1.725	317

Analyte18

Analyte Name:	B6
Analyte Description:	
Analyte ID:	18
Analyte Creation Date/Time:	2021-11-19T14:09:10.104
Applied MSP Library(ies):	
Applied Taxonomy Tree:	Projects, Bruker Taxonomy

Rank (Quality)	Matched Pattern	Score Value	NCBI Identifier
1 (++)	Pseudomonas savastanoi ssp savastanoi LMG 2209T HAM	2.238	29438
2 (++)	Pseudomonas savastanoi ssp savastanoi LMG 5011 HAM	2.125	29438
3 (++)	Pseudomonas caricapapayae LMG 2152T HAM	2	46678
4 (+)	Pseudomonas syringae ssp syringae DSM 6693 HAM	1.922	317
5 (+)	2501	1.865	451320
6 (+)	Pseudomonas congelans DSM 14939T HAM	1.844	200452
7 (+)	Pseudomonas avellanae DSM 11809T DSM	1.827	46257
8 (+)	Pseudomonas agarici DSM 11810T HAM	1.788	46677
9 (+)	Pseudomonas cichorii DSM 50259T HAM	1.786	36746
10 (+)	Pseudomonas chlororaphis ssp chlororaphis DSM 50083T HAM	1.761	333

Analyte19

Analyte Name:	B7
Analyte Description:	
Analyte ID:	19
Analyte Creation Date/Time:	2021-11-19T14:09:09.351
Applied MSP Library(ies):	
Applied Taxonomy Tree:	Projects, Bruker Taxonomy

Rank (Quality)	Matched Pattern	Score Value	NCBI Identifier
1 (++)	Pseudomonas savastanoi ssp savastanoi LMG 2209T HAM	2.294	29438
2 (++)	Pseudomonas savastanoi ssp savastanoi LMG 5011 HAM	2.214	29438
3 (+)	Pseudomonas caricapapayae LMG 2152T HAM	1.996	46678
4 (+)	Pseudomonas syringae ssp syringae DSM 6693 HAM	1.909	317
5 (+)	Pseudomonas congelans DSM 14939T HAM	1.84	200452
6 (+)	Pseudomonas avellanae DSM 11809T DSM	1.834	46257
7 (+)	Pseudomonas agarici DSM 11810T HAM	1.827	46677
8 (+)	2502	1.755	451320
9 (+)	2501	1.754	451320
10 (+)	Pseudomonas cichorii DSM 50259T HAM	1.733	36746

Analyte20

Analyte Name:	B8
Analyte Description:	
Analyte ID:	20
Analyte Creation Date/Time:	2021-11-19T14:09:09.309
Applied MSP Library(ies):	
Applied Taxonomy Tree:	Projects, Bruker Taxonomy

Rank (Quality)	Matched Pattern	Score Value	NCBI Identifier
1 (+++)	Pseudomonas savastanoi ssp savastanoi LMG 2209T HAM	2.37	29438
2 (++)	Pseudomonas savastanoi ssp savastanoi LMG 5011 HAM	2.228	29438
3 (++)	Pseudomonas caricapapayae LMG 2152T HAM	2.082	46678
4 (+)	Pseudomonas syringae ssp syringae DSM 6693 HAM	1.99	317
5 (+)	2502	1.979	451320
6 (+)	2501	1.95	451320
7 (+)	Pseudomonas avellanae DSM 11809T DSM	1.902	46257
8 (+)	Pseudomonas congelans DSM 14939T HAM	1.878	200452
9 (+)	Pseudomonas agarici DSM 11810T HAM	1.792	46677
10 (+)	Pseudomonas cichorii DSM 50259T HAM	1.759	36746

Analyte21

Analyte Name:	B9
Analyte Description:	
Analyte ID:	21
Analyte Creation Date/Time:	2021-11-19T14:09:10.213
Applied MSP Library(ies):	
Applied Taxonomy Tree:	Projects, Bruker Taxonomy

Rank (Quality)	Matched Pattern	Score Value	NCBI Identifier
1 (++)	Pseudomonas savastanoi ssp savastanoi LMG 2209T HAM	2.243	29438
2 (++)	Pseudomonas savastanoi ssp savastanoi LMG 5011 HAM	2.173	29438
3 (++)	Pseudomonas caricapapayae LMG 2152T HAM	2.013	46678
4 (+)	Pseudomonas syringae ssp syringae DSM 6693 HAM	1.947	317
5 (+)	Pseudomonas agarici DSM 11810T HAM	1.876	46677
6 (+)	Pseudomonas congelans DSM 14939T HAM	1.859	200452
7 (+)	Pseudomonas cichorii DSM 50259T HAM	1.784	36746
8 (+)	Pseudomonas chlororaphis ssp chlororaphis DSM 50083T HAM	1.764	333
9 (+)	2501	1.739	451320
10 (+)	Pseudomonas avellanae DSM 11809T DSM	1.712	46257

Analyte22

Analyte Name:	B10
Analyte Description:	
Analyte ID:	22
Analyte Creation Date/Time:	2021-11-19T14:09:10.475
Applied MSP Library(ies):	
Applied Taxonomy Tree:	Projects, Bruker Taxonomy

Rank (Quality)	Matched Pattern	Score Value	NCBI Identifier
1 (++)	Pseudomonas savastanoi ssp savastanoi LMG 2209T HAM	2.267	29438
2 (++)	Pseudomonas savastanoi ssp savastanoi LMG 5011 HAM	2.162	29438
3 (++)	Pseudomonas caricapapayae LMG 2152T HAM	2.003	46678
4 (+)	Pseudomonas syringae ssp syringae DSM 6693 HAM	1.924	317
5 (+)	Pseudomonas congelans DSM 14939T HAM	1.81	200452
6 (+)	Pseudomonas avellanae DSM 11809T DSM	1.758	46257
7 (+)	Pseudomonas agarici DSM 11810T HAM	1.754	46677
8 (+)	2501	1.719	451320
9 (+)	Pseudomonas chlororaphis ssp chlororaphis DSM 50083T HAM	1.712	333
10 (-)	Pseudomonas syringae ssp syringae LMG 1247T HAM	1.586	317

Analyte23

Analyte Name:	B11
Analyte Description:	
Analyte ID:	23
Analyte Creation Date/Time:	2021-11-19T14:09:10.506
Applied MSP Library(ies):	
Applied Taxonomy Tree:	Projects, Bruker Taxonomy

Rank (Quality)	Matched Pattern	Score Value	NCBI Identifier
1 (+++)	Pseudomonas savastanoi ssp savastanoi LMG 2209T HAM	2.383	29438
2 (++)	Pseudomonas savastanoi ssp savastanoi LMG 5011 HAM	2.143	29438
3 (+)	2501	1.953	451320
4 (+)	Pseudomonas caricapapayae LMG 2152T HAM	1.936	46678
5 (+)	Pseudomonas congelans DSM 14939T HAM	1.863	200452
6 (+)	Pseudomonas syringae ssp syringae DSM 6693 HAM	1.861	317
7 (+)	2502	1.849	451320
8 (+)	Pseudomonas avellanae DSM 11809T DSM	1.847	46257
9 (+)	Pseudomonas agarici DSM 11810T HAM	1.835	46677
10 (-)	Pseudomonas chlororaphis ssp aurantiaca CIP 106718T HAM	1.604	333

Analyte24

Analyte Name:	B12
Analyte Description:	
Analyte ID:	24
Analyte Creation Date/Time:	2021-11-19T14:09:10.969
Applied MSP Library(ies):	
Applied Taxonomy Tree:	Projects, Bruker Taxonomy

Rank (Quality)	Matched Pattern	Score Value	NCBI Identifier
1 (+++)	Pseudomonas savastanoi ssp savastanoi LMG 2209T HAM	2.308	29438
2 (++)	Pseudomonas savastanoi ssp savastanoi LMG 5011 HAM	2.194	29438
3 (+)	Pseudomonas syringae ssp syringae DSM 6693 HAM	1.929	317
4 (+)	Pseudomonas caricapapayae LMG 2152T HAM	1.919	46678
5 (+)	Pseudomonas agarici DSM 11810T HAM	1.909	46677
6 (+)	2501	1.848	451320
7 (+)	Pseudomonas congelans DSM 14939T HAM	1.803	200452
8 (+)	2502	1.794	451320
9 (+)	Pseudomonas syringae ssp syringae LMG 1247T HAM	1.73	317
10 (-)	Pseudomonas avellanae DSM 11809T DSM	1.649	46257

Analyte25

Analyte Name:	C1
Analyte Description:	
Analyte ID:	25
Analyte Creation Date/Time:	2021-11-19T14:09:10.406
Applied MSP Library(ies):	
Applied Taxonomy Tree:	Projects, Bruker Taxonomy

Rank (Quality)	Matched Pattern	Score Value	NCBI Identifier
1 (+++)	Pseudomonas savastanoi ssp savastanoi LMG 2209T HAM	2.314	29438
2 (++)	Pseudomonas savastanoi ssp savastanoi LMG 5011 HAM	2.246	29438
3 (+)	Pseudomonas syringae ssp syringae DSM 6693 HAM	1.986	317
4 (+)	Pseudomonas caricapapayae LMG 2152T HAM	1.975	46678
5 (+)	2501	1.962	451320
6 (+)	2502	1.86	451320
7 (+)	Pseudomonas congelans DSM 14939T HAM	1.779	200452
8 (+)	Pseudomonas viridiflava DSM 11124T HAM	1.753	33069
9 (+)	Pseudomonas syringae ssp syringae LMG 1247T HAM	1.733	317
10 (+)	Pseudomonas agarici DSM 11810T HAM	1.716	46677

Analyte26

Analyte Name:	C2
Analyte Description:	
Analyte ID:	26
Analyte Creation Date/Time:	2021-11-19T14:09:10.046
Applied MSP Library(ies):	
Applied Taxonomy Tree:	Projects, Bruker Taxonomy

Rank (Quality)	Matched Pattern	Score Value	NCBI Identifier
1 (++)	Pseudomonas savastanoi ssp savastanoi LMG 2209T HAM	2.289	29438
2 (++)	Pseudomonas savastanoi ssp savastanoi LMG 5011 HAM	2.095	29438
3 (+)	Pseudomonas caricapapayae LMG 2152T HAM	1.946	46678
4 (+)	2501	1.922	451320
5 (+)	Pseudomonas syringae ssp syringae DSM 6693 HAM	1.89	317
6 (+)	2502	1.843	451320
7 (+)	Pseudomonas congelans DSM 14939T HAM	1.843	200452
8 (+)	Pseudomonas agarici DSM 11810T HAM	1.81	46677
9 (+)	Pseudomonas avellanae DSM 11809T DSM	1.739	46257
10 (-)	Pseudomonas syringae ssp syringae LMG 1247T HAM	1.639	317

Analyte27

Analyte Name: C3
Analyte Description:
Analyte ID: 27
Analyte Creation Date/Time: 2021-11-19T14:09:10.171
Applied MSP Library(ies):
Applied Taxonomy Tree: Projects, Bruker Taxonomy

Rank (Quality)	Matched Pattern	Score Value	NCBI Identifier
1 (+++)	Pseudomonas savastanoi ssp savastanoi LMG 2209T HAM	2.32	29438
2 (++)	Pseudomonas savastanoi ssp savastanoi LMG 5011 HAM	2.188	29438
3 (+)	Pseudomonas caricapapayae LMG 2152T HAM	1.994	46678
4 (+)	2501	1.906	451320
5 (+)	Pseudomonas syringae ssp syringae DSM 6693 HAM	1.884	317
6 (+)	Pseudomonas congelans DSM 14939T HAM	1.881	200452
7 (+)	Pseudomonas agarici DSM 11810T HAM	1.873	46677
8 (+)	Pseudomonas avellanae DSM 11809T DSM	1.752	46257
9 (-)	2502	1.692	451320
10 (-)	Pseudomonas chlororaphis ssp chlororaphis DSM 50083T HAM	1.617	333

Analyte28

Analyte Name:	C4
Analyte Description:	
Analyte ID:	28
Analyte Creation Date/Time:	2021-11-19T14:09:10.565
Applied MSP Library(ies):	
Applied Taxonomy Tree:	Projects, Bruker Taxonomy

Rank (Quality)	Matched Pattern	Score Value	NCBI Identifier
1 (++)	Pseudomonas savastanoi ssp savastanoi LMG 2209T HAM	2.257	29438
2 (++)	Pseudomonas savastanoi ssp savastanoi LMG 5011 HAM	2.151	29438
3 (++)	Pseudomonas caricapapayae LMG 2152T HAM	2.025	46678
4 (+)	Pseudomonas syringae ssp syringae DSM 6693 HAM	1.967	317
5 (+)	2501	1.926	451320
6 (+)	2502	1.89	451320
7 (+)	Pseudomonas congelans DSM 14939T HAM	1.847	200452
8 (+)	Pseudomonas cichorii DSM 50259T HAM	1.812	36746
9 (+)	Pseudomonas agarici DSM 11810T HAM	1.736	46677
10 (+)	Pseudomonas avellanae DSM 11809T DSM	1.721	46257

Analyte29

Analyte Name:	C5
Analyte Description:	
Analyte ID:	29
Analyte Creation Date/Time:	2021-11-19T14:09:10.260
Applied MSP Library(ies):	
Applied Taxonomy Tree:	Projects, Bruker Taxonomy

Rank (Quality)	Matched Pattern	Score Value	NCBI Identifier
1 (++)	Pseudomonas savastanoi ssp savastanoi LMG 2209T HAM	2.291	29438
2 (++)	Pseudomonas savastanoi ssp savastanoi LMG 5011 HAM	2.183	29438
3 (+)	Pseudomonas caricapapayae LMG 2152T HAM	1.977	46678
4 (+)	2501	1.932	451320
5 (+)	2502	1.914	451320
6 (+)	Pseudomonas syringae ssp syringae DSM 6693 HAM	1.823	317
7 (+)	Pseudomonas congelans DSM 14939T HAM	1.808	200452
8 (+)	Pseudomonas chlororaphis ssp chlororaphis DSM 50083T HAM	1.798	333
9 (+)	Pseudomonas agarici DSM 11810T HAM	1.769	46677
10 (+)	Pseudomonas avellanae DSM 11809T DSM	1.733	46257

Analyte30

Analyte Name:	C6
Analyte Description:	
Analyte ID:	30
Analyte Creation Date/Time:	2021-11-19T14:09:10.792
Applied MSP Library(ies):	
Applied Taxonomy Tree:	Projects, Bruker Taxonomy

Rank (Quality)	Matched Pattern	Score Value	NCBI Identifier
1 (++)	Pseudomonas savastanoi ssp savastanoi LMG 2209T HAM	2.297	29438
2 (++)	Pseudomonas savastanoi ssp savastanoi LMG 5011 HAM	2.249	29438
3 (++)	Pseudomonas caricapapayae LMG 2152T HAM	2.027	46678
4 (+)	2501	1.886	451320
5 (+)	Pseudomonas syringae ssp syringae DSM 6693 HAM	1.869	317
6 (+)	2502	1.85	451320
7 (+)	Pseudomonas congelans DSM 14939T HAM	1.848	200452
8 (+)	Pseudomonas chlororaphis ssp chlororaphis DSM 50083T HAM	1.817	333
9 (+)	Pseudomonas avellanae DSM 11809T DSM	1.814	46257
10 (+)	Pseudomonas chlororaphis ssp aurantiaca CIP 106718T HAM	1.756	333

Analyte31

Analyte Name:	C7
Analyte Description:	
Analyte ID:	31
Analyte Creation Date/Time:	2021-11-19T14:09:10.121
Applied MSP Library(ies):	
Applied Taxonomy Tree:	Projects, Bruker Taxonomy

Rank (Quality)	Matched Pattern	Score Value	NCBI Identifier
1 (++)	Pseudomonas savastanoi ssp savastanoi LMG 5011 HAM	2.276	29438
2 (++)	Pseudomonas savastanoi ssp savastanoi LMG 2209T HAM	2.235	29438
3 (+)	Pseudomonas caricapapayae LMG 2152T HAM	1.977	46678
4 (+)	2501	1.959	451320
5 (+)	Pseudomonas syringae ssp syringae DSM 6693 HAM	1.941	317
6 (+)	2502	1.913	451320
7 (+)	Pseudomonas avellanae DSM 11809T DSM	1.875	46257
8 (+)	Pseudomonas congelans DSM 14939T HAM	1.772	200452
9 (+)	Pseudomonas agarici DSM 11810T HAM	1.76	46677
10 (+)	Pseudomonas syringae ssp syringae LMG 1247T HAM	1.729	317

Analyte32

Analyte Name:	C8
Analyte Description:	
Analyte ID:	32
Analyte Creation Date/Time:	2021-11-19T14:09:10.347
Applied MSP Library(ies):	
Applied Taxonomy Tree:	Projects, Bruker Taxonomy

Rank (Quality)	Matched Pattern	Score Value	NCBI Identifier
1 (++)	Pseudomonas savastanoi ssp savastanoi LMG 2209T HAM	2.297	29438
2 (++)	Pseudomonas savastanoi ssp savastanoi LMG 5011 HAM	2.283	29438
3 (++)	Pseudomonas caricapapayae LMG 2152T HAM	2.071	46678
4 (+)	Pseudomonas syringae ssp syringae DSM 6693 HAM	1.936	317
5 (+)	Pseudomonas congelans DSM 14939T HAM	1.905	200452
6 (+)	2502	1.894	451320
7 (+)	2501	1.845	451320
8 (+)	Pseudomonas avellanae DSM 11809T DSM	1.766	46257
9 (+)	Pseudomonas agarici DSM 11810T HAM	1.705	46677
10 (-)	Pseudomonas chlororaphis ssp chlororaphis DSM 50083T HAM	1.696	333

Analyte33

Analyte Name:	C9
Analyte Description:	
Analyte ID:	33
Analyte Creation Date/Time:	2021-11-19T14:09:09.300
Applied MSP Library(ies):	
Applied Taxonomy Tree:	Projects, Bruker Taxonomy

Rank (Quality)	Matched Pattern	Score Value	NCBI Identifier
1 (+++)	Pseudomonas savastanoi ssp savastanoi LMG 2209T HAM	2.334	29438
2 (++)	Pseudomonas savastanoi ssp savastanoi LMG 5011 HAM	2.238	29438
3 (++)	Pseudomonas caricapapayae LMG 2152T HAM	2.043	46678
4 (+)	Pseudomonas syringae ssp syringae DSM 6693 HAM	1.922	317
5 (+)	Pseudomonas congelans DSM 14939T HAM	1.905	200452
6 (+)	Pseudomonas agarici DSM 11810T HAM	1.841	46677
7 (+)	2501	1.815	451320
8 (+)	2502	1.799	451320
9 (+)	Pseudomonas chlororaphis ssp chlororaphis DSM 50083T HAM	1.774	333
10 (+)	Pseudomonas cichorii DSM 50259T HAM	1.753	36746

Analyte34

Analyte Name:	C10
Analyte Description:	
Analyte ID:	34
Analyte Creation Date/Time:	2021-11-19T14:09:10.288
Applied MSP Library(ies):	
Applied Taxonomy Tree:	Projects, Bruker Taxonomy

Rank (Quality)	Matched Pattern	Score Value	NCBI Identifier
1 (++)	Pseudomonas savastanoi ssp savastanoi LMG 2209T HAM	2.178	29438
2 (++)	Pseudomonas savastanoi ssp savastanoi LMG 5011 HAM	2.143	29438
3 (+)	2501	1.957	451320
4 (+)	Pseudomonas caricapapayae LMG 2152T HAM	1.948	46678
5 (+)	2502	1.845	451320
6 (+)	Pseudomonas congelans DSM 14939T HAM	1.787	200452
7 (+)	Pseudomonas syringae ssp syringae DSM 6693 HAM	1.777	317
8 (+)	Pseudomonas agarici DSM 11810T HAM	1.753	46677
9 (-)	Pseudomonas cichorii DSM 50259T HAM	1.678	36746
10 (-)	Pseudomonas chlororaphis ssp chlororaphis DSM 50083T HAM	1.631	333

Analyte35

Analyte Name:	C11
Analyte Description:	
Analyte ID:	35
Analyte Creation Date/Time:	2021-11-19T14:09:09.644
Applied MSP Library(ies):	
Applied Taxonomy Tree:	Bruker Taxonomy, Projects

Rank (Quality)	Matched Pattern	Score Value	NCBI Identifier
1 (++)	Pseudomonas savastanoi ssp savastanoi LMG 2209T HAM	2.291	29438
2 (++)	Pseudomonas savastanoi ssp savastanoi LMG 5011 HAM	2.167	29438
3 (++)	Pseudomonas caricapapayae LMG 2152T HAM	2.034	46678
4 (+)	Pseudomonas syringae ssp syringae DSM 6693 HAM	1.951	317
5 (+)	2501	1.894	451320
6 (+)	Pseudomonas congelans DSM 14939T HAM	1.872	200452
7 (+)	2502	1.843	451320
8 (+)	Pseudomonas agarici DSM 11810T HAM	1.77	46677
9 (+)	Pseudomonas graminis DSM 11363T HAM	1.713	158627
10 (-)	Pseudomonas syringae ssp syringae LMG 1247T HAM	1.688	317

Analyte36

Analyte Name:	C12
Analyte Description:	
Analyte ID:	36
Analyte Creation Date/Time:	2021-11-19T14:09:09.299
Applied MSP Library(ies):	
Applied Taxonomy Tree:	Projects, Bruker Taxonomy

Rank (Quality)	Matched Pattern	Score Value	NCBI Identifier
1 (++)	Pseudomonas savastanoi ssp savastanoi LMG 2209T HAM	2.26	29438
2 (++)	Pseudomonas savastanoi ssp savastanoi LMG 5011 HAM	2.169	29438
3 (+)	Pseudomonas syringae ssp syringae DSM 6693 HAM	1.898	317
4 (+)	2501	1.86	451320
5 (+)	Pseudomonas caricapapayae LMG 2152T HAM	1.834	46678
6 (+)	Pseudomonas agarici DSM 11810T HAM	1.798	46677
7 (+)	Pseudomonas congelans DSM 14939T HAM	1.771	200452
8 (-)	Pseudomonas avellanae DSM 11809T DSM	1.681	46257
9 (-)	Pseudomonas syringae ssp syringae LMG 1247T HAM	1.625	317
10 (-)	Pseudomonas japonica DSM 22348T DSM	1.612	256466

Analyte37

Analyte Name:	D1
Analyte Description:	
Analyte ID:	37
Analyte Creation Date/Time:	2021-11-19T14:09:10.052
Applied MSP Library(ies):	
Applied Taxonomy Tree:	Projects, Bruker Taxonomy

Rank (Quality)	Matched Pattern	Score Value	NCBI Identifier
1 **(-)**	**no peaks found**	< 0	-
2 **(-)**	**no peaks found**	< 0	-
3 **(-)**	**no peaks found**	< 0	-
4 **(-)**	**no peaks found**	< 0	-
5 **(-)**	**no peaks found**	< 0	-
6 **(-)**	**no peaks found**	< 0	-
7 **(-)**	**no peaks found**	< 0	-
8 **(-)**	**no peaks found**	< 0	-
9 **(-)**	**no peaks found**	< 0	-
10 **(-)**	**no peaks found**	< 0	-

Analyte38

Analyte Name:	D2
Analyte Description:	
Analyte ID:	38
Analyte Creation Date/Time:	2021-11-19T14:09:10.843
Applied MSP Library(ies):	
Applied Taxonomy Tree:	Projects, Bruker Taxonomy

Rank (Quality)	Matched Pattern	Score Value	NCBI Identifier
1 (++)	Pseudomonas savastanoi ssp savastanoi LMG 2209T HAM	2.265	29438
2 (++)	Pseudomonas savastanoi ssp savastanoi LMG 5011 HAM	2.183	29438
3 (++)	Pseudomonas caricapapayae LMG 2152T HAM	2.088	46678
4 (+)	2501	1.953	451320
5 (+)	2502	1.92	451320
6 (+)	Pseudomonas syringae ssp syringae DSM 6693 HAM	1.846	317
7 (+)	Pseudomonas avellanae DSM 11809T DSM	1.844	46257
8 (+)	Pseudomonas congelans DSM 14939T HAM	1.773	200452
9 (+)	Pseudomonas chlororaphis ssp chlororaphis DSM 50083T HAM	1.727	333
10 (+)	Pseudomonas agarici DSM 11810T HAM	1.721	46677

Analyte39

Analyte Name: D3
Analyte Description:
Analyte ID: 39
Analyte Creation Date/Time: 2021-11-19T14:09:10.255
Applied MSP Library(ies):
Applied Taxonomy Tree: Projects, Bruker Taxonomy

Rank (Quality)	Matched Pattern	Score Value	NCBI Identifier
1 (++)	Pseudomonas savastanoi ssp savastanoi LMG 2209T HAM	2.256	29438
2 (++)	Pseudomonas savastanoi ssp savastanoi LMG 5011 HAM	2.123	29438
3 (++)	Pseudomonas caricapapayae LMG 2152T HAM	2.026	46678
4 (++)	2501	2	451320
5 (+)	Pseudomonas avellanae DSM 11809T DSM	1.9	46257
6 (+)	Pseudomonas syringae ssp syringae DSM 6693 HAM	1.898	317
7 (+)	Pseudomonas congelans DSM 14939T HAM	1.872	200452
8 (+)	2502	1.848	451320
9 (+)	Pseudomonas agarici DSM 11810T HAM	1.784	46677
10 (-)	Pseudomonas chlororaphis ssp aurantiaca CIP 106718T HAM	1.632	333

Analyte40

Analyte Name:	D4
Analyte Description:	
Analyte ID:	40
Analyte Creation Date/Time:	2021-11-19T14:09:09.919
Applied MSP Library(ies):	
Applied Taxonomy Tree:	Projects, Bruker Taxonomy

Rank (Quality)	Matched Pattern	Score Value	NCBI Identifier
1 (++)	Pseudomonas savastanoi ssp savastanoi LMG 2209T HAM	2.278	29438
2 (++)	Pseudomonas savastanoi ssp savastanoi LMG 5011 HAM	2.219	29438
3 (++)	2501	2.045	451320
4 (++)	Pseudomonas caricapapayae LMG 2152T HAM	2.032	46678
5 (+)	2502	1.932	451320
6 (+)	Pseudomonas congelans DSM 14939T HAM	1.85	200452
7 (+)	Pseudomonas syringae ssp syringae DSM 6693 HAM	1.833	317
8 (+)	Pseudomonas avellanae DSM 11809T DSM	1.805	46257
9 (+)	Pseudomonas chlororaphis ssp chlororaphis DSM 50083T HAM	1.767	333
10 (+)	Pseudomonas chlororaphis ssp aurantiaca CIP 106718T HAM	1.761	333

Analyte41

Analyte Name:	D5
Analyte Description:	
Analyte ID:	41
Analyte Creation Date/Time:	2021-11-19T14:09:10.465
Applied MSP Library(ies):	
Applied Taxonomy Tree:	Projects, Bruker Taxonomy

Rank (Quality)	Matched Pattern	Score Value	NCBI Identifier
1 (+++)	Pseudomonas savastanoi ssp savastanoi LMG 2209T HAM	2.369	29438
2 (++)	Pseudomonas savastanoi ssp savastanoi LMG 5011 HAM	2.115	29438
3 (++)	Pseudomonas caricapapayae LMG 2152T HAM	2.019	46678
4 (+)	2501	1.891	451320
5 (+)	Pseudomonas congelans DSM 14939T HAM	1.888	200452
6 (+)	2502	1.859	451320
7 (+)	Pseudomonas syringae ssp syringae DSM 6693 HAM	1.857	317
8 (+)	Pseudomonas agarici DSM 11810T HAM	1.769	46677
9 (+)	Pseudomonas avellanae DSM 11809T DSM	1.76	46257
10 (+)	Pseudomonas chlororaphis ssp aurantiaca CIP 106718T HAM	1.708	333

Analyte42

Analyte Name:	D6
Analyte Description:	
Analyte ID:	42
Analyte Creation Date/Time:	2021-11-19T14:09:09.833
Applied MSP Library(ies):	
Applied Taxonomy Tree:	Projects, Bruker Taxonomy

Rank (Quality)	Matched Pattern	Score Value	NCBI Identifier
1 (++)	Pseudomonas savastanoi ssp savastanoi LMG 2209T HAM	2.283	29438
2 (++)	Pseudomonas savastanoi ssp savastanoi LMG 5011 HAM	2.061	29438
3 (+)	Pseudomonas caricapapayae LMG 2152T HAM	1.973	46678
4 (+)	2501	1.957	451320
5 (+)	2502	1.939	451320
6 (+)	Pseudomonas avellanae DSM 11809T DSM	1.908	46257
7 (+)	Pseudomonas congelans DSM 14939T HAM	1.839	200452
8 (+)	Pseudomonas syringae ssp syringae DSM 6693 HAM	1.81	317
9 (+)	Pseudomonas agarici DSM 11810T HAM	1.808	46677
10 (+)	Pseudomonas chlororaphis ssp aurantiaca CIP 106718T HAM	1.711	333

Analyte43

Analyte Name:	D7
Analyte Description:	
Analyte ID:	43
Analyte Creation Date/Time:	2021-11-19T14:09:10.940
Applied MSP Library(ies):	
Applied Taxonomy Tree:	Bruker Taxonomy, Projects

Rank (Quality)	Matched Pattern	Score Value	NCBI Identifier
1 (+++)	Pseudomonas savastanoi ssp savastanoi LMG 2209T HAM	2.351	29438
2 (++)	Pseudomonas savastanoi ssp savastanoi LMG 5011 HAM	2.183	29438
3 (++)	Pseudomonas caricapapayae LMG 2152T HAM	2.026	46678
4 (+)	Pseudomonas syringae ssp syringae DSM 6693 HAM	1.952	317
5 (+)	Pseudomonas congelans DSM 14939T HAM	1.904	200452
6 (+)	2502	1.852	451320
7 (+)	2501	1.836	451320
8 (+)	Pseudomonas agarici DSM 11810T HAM	1.782	46677
9 (+)	Pseudomonas viridiflava DSM 11124T HAM	1.743	33069
10 (+)	Pseudomonas chlororaphis ssp chlororaphis DSM 50083T HAM	1.734	333

Analyte44

BRUKER

Analyte Name:	D8
Analyte Description:	
Analyte ID:	44
Analyte Creation Date/Time:	2021-11-19T14:09:10.767
Applied MSP Library(ies):	
Applied Taxonomy Tree:	Projects, Bruker Taxonomy

Rank (Quality)	Matched Pattern	Score Value	NCBI Identifier
1 (+++)	Pseudomonas savastanoi ssp savastanoi LMG 2209T HAM	2.347	29438
2 (++)	Pseudomonas savastanoi ssp savastanoi LMG 5011 HAM	2.173	29438
3 (++)	Pseudomonas caricapapayae LMG 2152T HAM	2.008	46678
4 (+)	2502	1.878	451320
5 (+)	Pseudomonas syringae ssp syringae DSM 6693 HAM	1.854	317
6 (+)	Pseudomonas congelans DSM 14939T HAM	1.844	200452
7 (+)	Pseudomonas agarici DSM 11810T HAM	1.807	46677
8 (+)	2501	1.787	451320
9 (+)	Pseudomonas avellanae DSM 11809T DSM	1.724	46257
10 (-)	Pseudomonas chlororaphis ssp chlororaphis DSM 50083T HAM	1.67	333

Analyte45

Analyte Name:	D9
Analyte Description:	
Analyte ID:	45
Analyte Creation Date/Time:	2021-11-19T14:09:09.894
Applied MSP Library(ies):	
Applied Taxonomy Tree:	Bruker Taxonomy, Projects

Rank (Quality)	Matched Pattern	Score Value	NCBI Identifier
1 (++)	Pseudomonas savastanoi ssp savastanoi LMG 2209T HAM	2.228	29438
2 (++)	Pseudomonas savastanoi ssp savastanoi LMG 5011 HAM	2.113	29438
3 (++)	Pseudomonas caricapapayae LMG 2152T HAM	2.034	46678
4 (+)	2501	1.995	451320
5 (+)	Pseudomonas congelans DSM 14939T HAM	1.834	200452
6 (+)	2502	1.83	451320
7 (+)	Pseudomonas syringae ssp syringae DSM 6693 HAM	1.772	317
8 (+)	Pseudomonas chlororaphis ssp chlororaphis DSM 50083T HAM	1.711	333
9 (+)	Pseudomonas cichorii DSM 50259T HAM	1.706	36746
10 (+)	Pseudomonas avellanae DSM 11809T DSM	1.705	46257

Analyte46

Analyte Name: D10
Analyte Description:
Analyte ID: 46
Analyte Creation Date/Time: 2021-11-19T14:09:09.787
Applied MSP Library(ies):
Applied Taxonomy Tree: Projects, Bruker Taxonomy

Rank (Quality)	Matched Pattern	Score Value	NCBI Identifier
1 (+++)	Pseudomonas savastanoi ssp savastanoi LMG 2209T HAM	2.357	29438
2 (++)	Pseudomonas savastanoi ssp savastanoi LMG 5011 HAM	2.168	29438
3 (++)	Pseudomonas caricapapayae LMG 2152T HAM	2.08	46678
4 (++)	2501	2.009	451320
5 (+)	2502	1.95	451320
6 (+)	Pseudomonas congelans DSM 14939T HAM	1.856	200452
7 (+)	Pseudomonas agarici DSM 11810T HAM	1.825	46677
8 (+)	Pseudomonas avellanae DSM 11809T DSM	1.809	46257
9 (+)	Pseudomonas syringae ssp syringae DSM 6693 HAM	1.8	317
10 (-)	Pseudomonas cichorii DSM 50259T HAM	1.698	36746

Analyte47

Analyte Name:	D11
Analyte Description:	
Analyte ID:	47
Analyte Creation Date/Time:	2021-11-19T14:09:09.411
Applied MSP Library(ies):	
Applied Taxonomy Tree:	Projects, Bruker Taxonomy

Rank (Quality)	Matched Pattern	Score Value	NCBI Identifier
1 (+++)	Pseudomonas savastanoi ssp savastanoi LMG 2209T HAM	2.325	29438
2 (++)	Pseudomonas savastanoi ssp savastanoi LMG 5011 HAM	2.087	29438
3 (+)	Pseudomonas caricapapayae LMG 2152T HAM	1.965	46678
4 (+)	2501	1.955	451320
5 (+)	Pseudomonas avellanae DSM 11809T DSM	1.917	46257
6 (+)	Pseudomonas syringae ssp syringae DSM 6693 HAM	1.853	317
7 (+)	Pseudomonas agarici DSM 11810T HAM	1.842	46677
8 (+)	Pseudomonas congelans DSM 14939T HAM	1.824	200452
9 (+)	2502	1.817	451320
10 (-)	Pseudomonas chlororaphis ssp chlororaphis DSM 50083T HAM	1.692	333

Analyte48

Analyte Name:	D12
Analyte Description:	
Analyte ID:	48
Analyte Creation Date/Time:	2021-11-19T14:09:10.708
Applied MSP Library(ies):	
Applied Taxonomy Tree:	Projects, Bruker Taxonomy

Rank (Quality)	Matched Pattern	Score Value	NCBI Identifier
1 (+++)	Pseudomonas savastanoi ssp savastanoi LMG 2209T HAM	2.321	29438
2 (++)	Pseudomonas savastanoi ssp savastanoi LMG 5011 HAM	2.078	29438
3 (++)	Pseudomonas caricapapayae LMG 2152T HAM	2.033	46678
4 (+)	2501	1.989	451320
5 (+)	Pseudomonas syringae ssp syringae DSM 6693 HAM	1.916	317
6 (+)	2502	1.882	451320
7 (+)	Pseudomonas congelans DSM 14939T HAM	1.882	200452
8 (+)	Pseudomonas avellanae DSM 11809T DSM	1.845	46257
9 (+)	Pseudomonas agarici DSM 11810T HAM	1.844	46677
10 (-)	Pseudomonas chlororaphis ssp chlororaphis DSM 50083T HAM	1.682	333

Analyte49

Analyte Name: E1
Analyte Description:
Analyte ID: 49
Analyte Creation Date/Time: 2021-11-19T14:09:09.568
Applied MSP Library(ies):
Applied Taxonomy Tree: Projects, Bruker Taxonomy

Rank (Quality)	Matched Pattern	Score Value	NCBI Identifier
1 (++)	Pseudomonas savastanoi ssp savastanoi LMG 2209T HAM	2.289	29438
2 (++)	Pseudomonas savastanoi ssp savastanoi LMG 5011 HAM	2.092	29438
3 (+)	2501	1.925	451320
4 (+)	Pseudomonas syringae ssp syringae DSM 6693 HAM	1.904	317
5 (+)	2502	1.886	451320
6 (+)	Pseudomonas congelans DSM 14939T HAM	1.859	200452
7 (+)	Pseudomonas caricapapayae LMG 2152T HAM	1.833	46678
8 (+)	Pseudomonas avellanae DSM 11809T DSM	1.769	46257
9 (+)	Pseudomonas agarici DSM 11810T HAM	1.761	46677
10 (-)	Pseudomonas oleovorans ssp oleovorans DSM 50188 HAM	1.647	301

Analyte50

Analyte Name:	E2
Analyte Description:	
Analyte ID:	50
Analyte Creation Date/Time:	2021-11-19T14:09:10.640
Applied MSP Library(ies):	
Applied Taxonomy Tree:	Projects, Bruker Taxonomy

Rank (Quality)	Matched Pattern	Score Value	NCBI Identifier
1 (-)	Curtobacterium flaccumfaciens DSM 20149 DSM	1.599	2035
2 (-)	Curtobacterium albidum HKI 11500 HKJ	1.377	50728
3 (-)	Sphingobium cloacae DSM 14926T HAM	1.278	120107
4 (-)	Staphylococcus lugdunensis DSM 4806 DSM	1.216	28035
5 (-)	Paenibacillus chinjuensis DSM 15045T DSM	1.196	103815
6 (-)	Starkeya novella B351 UFL	1.186	921
7 (-)	Arthrobacter psychrolactophilus DSM 15612T DSM	1.184	92442
8 (-)	Halococcus morrhuae B270 UFL	1.182	2250
9 (-)	Arthrobacter gandavensis DSM 15046T DSM	1.182	169960
10 (-)	Agromyces cerinus ssp nitratus HKI 11532_DSM 8596T HKJ	1.173	231391

Analyte51

Analyte Name:	E3
Analyte Description:	
Analyte ID:	51
Analyte Creation Date/Time:	2021-11-19T14:09:10.079
Applied MSP Library(ies):	
Applied Taxonomy Tree:	Projects, Bruker Taxonomy

Rank (Quality)	Matched Pattern	Score Value	NCBI Identifier
1 (+++)	Pseudomonas savastanoi ssp savastanoi LMG 2209T HAM	2.361	29438
2 (++)	Pseudomonas savastanoi ssp savastanoi LMG 5011 HAM	2.181	29438
3 (+)	Pseudomonas syringae ssp syringae DSM 6693 HAM	1.923	317
4 (+)	2501	1.922	451320
5 (+)	Pseudomonas caricapapayae LMG 2152T HAM	1.9	46678
6 (+)	Pseudomonas avellanae DSM 11809T DSM	1.849	46257
7 (+)	Pseudomonas congelans DSM 14939T HAM	1.832	200452
8 (+)	2502	1.83	451320
9 (+)	Pseudomonas agarici DSM 11810T HAM	1.782	46677
10 (-)	Pseudomonas cichorii DSM 50259T HAM	1.628	36746

Analyte52

Analyte Name:	E4
Analyte Description:	
Analyte ID:	52
Analyte Creation Date/Time:	2021-11-19T14:09:09.669
Applied MSP Library(ies):	
Applied Taxonomy Tree:	Projects, Bruker Taxonomy

Rank (Quality)	Matched Pattern	Score Value	NCBI Identifier
1 (-)	Curtobacterium flaccumfaciens DSM 20149 DSM	1.604	2035
2 (-)	Curtobacterium albidum HKI 11500 HKJ	1.502	50728
3 (-)	Paeniclostridium sordellii 1070_ATCC 9714T BOG	1.273	1505
4 (-)	Agromyces humatus DSM 16389T DSM	1.23	279573
5 (-)	Arthrobacter psychrolactophilus DSM 15612T DSM	1.229	92442
6 (-)	Lactobacillus sharpeae DSM 20506 DSM	1.219	1626
7 (-)	Lactobacillus amylovorus DSM 20531T DSM	1.21	1604
8 (-)	Lactobacillus paracasei ssp tolerans DSM 20012 DSM	1.209	113557
9 (-)	Staphylococcus lugdunensis DSM 4806 DSM	1.185	28035
10 (-)	Rhizobium radiobacter B167 UFL	1.172	358

Analyte53

Analyte Name: E5
Analyte Description:
Analyte ID: 53
Analyte Creation Date/Time: 2021-11-19T14:09:10.599
Applied MSP Library(ies):
Applied Taxonomy Tree: Projects, Bruker Taxonomy

Rank (Quality)	Matched Pattern	Score Value	NCBI Identifier
1 (-)	Curtobacterium flaccumfaciens DSM 20149 DSM	1.688	2035
2 (-)	Agromyces humatus DSM 16389T DSM	1.304	279573
3 (-)	Lactobacillus plantarum DSM 20246 DSM	1.27	1590
4 (-)	Arthrobacter gandavensis DSM 15046T DSM	1.242	169960
5 (-)	Curtobacterium albidum HKI 11500 HKJ	1.232	50728
6 (-)	Agromyces italicus HKI 325_DSM 16388T HKJ	1.215	279572
7 (-)	Chryseobacterium vrystaatense DSM 25206T DSM	1.196	307480
8 (-)	Citrobacter freundii 22054_1 CHB	1.183	546
9 (-)	Paraclostridium bifermentans 2273_CCUG 35297 BOG	1.175	1490
10 (-)	Lactobacillus paracasei ssp paracasei DSM 20244 DSM	1.172	47714

Analyte54

Analyte Name:	E6
Analyte Description:	
Analyte ID:	54
Analyte Creation Date/Time:	2021-11-19T14:09:10.322
Applied MSP Library(ies):	
Applied Taxonomy Tree:	Projects, Bruker Taxonomy

Rank (Quality)	Matched Pattern	Score Value	NCBI Identifier
1 (+++)	Bacillus megaterium DSM 1668 DSM	2.391	1404
2 (+++)	Bacillus megaterium DSM 2894 DSM	2.341	1404
3 (+++)	Bacillus megaterium DSM 32T DSM	2.326	1404
4 (-)	Bacillus sonorensis DSM 13779T DSM	1.498	119858
5 (-)	Rhizobium radiobacter B170 UFL	1.434	358
6 (-)	Cryptococcus gattii RV54130 CBS	1.294	292817
7 (-)	Mycobacterium pyrenivorans DSM 44605T DSM b	1.285	127740795
8 (-)	Lactobacillus vini DSM 20605T DSM	1.283	238015
9 (-)	Pseudarthrobacter oxydans DSM 20119T DSM	1.268	1671
10 (-)	Bacillus cohnii DSM 6307T DSM	1.245	33932

Analyte55

Analyte Name:	E7
Analyte Description:	
Analyte ID:	55
Analyte Creation Date/Time:	2021-11-19T14:09:09.493
Applied MSP Library(ies):	
Applied Taxonomy Tree:	Projects, Bruker Taxonomy

Rank (Quality)	Matched Pattern	Score Value	NCBI Identifier
1 (-)	Curtobacterium flaccumfaciens DSM 20149 DSM	1.642	2035
2 (-)	Curtobacterium albidum HKI 11500 HKJ	1.253	50728
3 (-)	Enterococcus faecium 11037 CHB	1.248	1352
4 (-)	Lactobacillus paracasei ssp tolerans DSM 20012 DSM	1.234	113557
5 (-)	Arthrobacter psychrolactophilus DSM 15612T DSM	1.189	92442
6 (-)	Citrobacter freundii 22054_1 CHB	1.183	546
7 (-)	Proteus vulgaris (PX) 22086129 MLD	1.152	585
8 (-)	Lactobacillus paracasei ssp paracasei DSM 8742 DSM	1.152	47714
9 (-)	Lactobacillus paracasei ssp paracasei DSM 20244 DSM	1.145	47714
10 (-)	Clostridium botulinum B 2293_NewZealand 75-3074_BOG	1.137	129286527

Analyte56

Analyte Name:	E8
Analyte Description:	
Analyte ID:	56
Analyte Creation Date/Time:	2021-11-19T14:09:10.456
Applied MSP Library(ies):	
Applied Taxonomy Tree:	Projects, Bruker Taxonomy

Rank (Quality)	Matched Pattern	Score Value	NCBI Identifier
1 (++)	Pseudomonas savastanoi ssp savastanoi LMG 2209T HAM	2.292	29438
2 (++)	Pseudomonas savastanoi ssp savastanoi LMG 5011 HAM	2.062	29438
3 (++)	Pseudomonas caricapapayae LMG 2152T HAM	2.021	46678
4 (+)	2501	1.932	451320
5 (+)	Pseudomonas avellanae DSM 11809T DSM	1.911	46257
6 (+)	Pseudomonas congelans DSM 14939T HAM	1.901	200452
7 (+)	Pseudomonas syringae ssp syringae DSM 6693 HAM	1.872	317
8 (+)	2502	1.836	451320
9 (+)	Pseudomonas chlororaphis ssp aurantiaca CIP 106718T HAM	1.809	333
10 (+)	Pseudomonas agarici DSM 11810T HAM	1.802	46677

Analyte57

Analyte Name:	E9
Analyte Description:	
Analyte ID:	57
Analyte Creation Date/Time:	2021-11-19T14:09:10.742
Applied MSP Library(ies):	
Applied Taxonomy Tree:	Projects, Bruker Taxonomy

Rank (Quality)	Matched Pattern	Score Value	NCBI Identifier
1 (+++)	Pseudomonas savastanoi ssp savastanoi LMG 2209T HAM	2.304	29438
2 (++)	Pseudomonas savastanoi ssp savastanoi LMG 5011 HAM	2.127	29438
3 (++)	Pseudomonas caricapapayae LMG 2152T HAM	2.089	46678
4 (+)	Pseudomonas congelans DSM 14939T HAM	1.922	200452
5 (+)	2501	1.897	451320
6 (+)	2502	1.883	451320
7 (+)	Pseudomonas syringae ssp syringae DSM 6693 HAM	1.858	317
8 (+)	Pseudomonas agarici DSM 11810T HAM	1.807	46677
9 (+)	Pseudomonas avellanae DSM 11809T DSM	1.711	46257
10 (+)	Pseudomonas chlororaphis ssp chlororaphis DSM 50083T HAM	1.708	333

Analyte58

Analyte Name: E10
Analyte Description:
Analyte ID: 58
Analyte Creation Date/Time: 2021-11-19T14:09:09.443
Applied MSP Library(ies):
Applied Taxonomy Tree: Projects, Bruker Taxonomy

Rank (Quality)	Matched Pattern	Score Value	NCBI Identifier
1 (+++)	Pseudomonas savastanoi ssp savastanoi LMG 2209T HAM	2.3	29438
2 (++)	Pseudomonas savastanoi ssp savastanoi LMG 5011 HAM	2.141	29438
3 (++)	Pseudomonas caricapapayae LMG 2152T HAM	2.033	46678
4 (+)	Pseudomonas syringae ssp syringae DSM 6693 HAM	1.939	317
5 (+)	2501	1.89	451320
6 (+)	Pseudomonas congelans DSM 14939T HAM	1.867	200452
7 (+)	2502	1.833	451320
8 (+)	Pseudomonas agarici DSM 11810T HAM	1.791	46677
9 (+)	Pseudomonas cichorii DSM 50259T HAM	1.777	36746
10 (+)	Pseudomonas avellanae DSM 11809T DSM	1.771	46257

Analyte59

Analyte Name:	E11
Analyte Description:	
Analyte ID:	59
Analyte Creation Date/Time:	2021-11-19T14:09:10.004
Applied MSP Library(ies):	
Applied Taxonomy Tree:	Bruker Taxonomy, Projects

Rank (Quality)	Matched Pattern	Score Value	NCBI Identifier
1 (++)	Pseudomonas savastanoi ssp savastanoi LMG 2209T HAM	2.244	29438
2 (++)	Pseudomonas caricapapayae LMG 2152T HAM	2.02	46678
3 (++)	Pseudomonas savastanoi ssp savastanoi LMG 5011 HAM	2.004	29438
4 (+)	2501	1.986	451320
5 (+)	2502	1.979	451320
6 (+)	Pseudomonas agarici DSM 11810T HAM	1.867	46677
7 (+)	Pseudomonas congelans DSM 14939T HAM	1.778	200452
8 (+)	Pseudomonas syringae ssp syringae DSM 6693 HAM	1.762	317
9 (+)	Pseudomonas chlororaphis ssp chlororaphis DSM 50083T HAM	1.736	333
10 (+)	Pseudomonas chlororaphis ssp aurantiaca CIP 106718T HAM	1.728	333

Analyte60

Analyte Name:	E12
Analyte Description:	
Analyte ID:	60
Analyte Creation Date/Time:	2021-11-19T14:09:10.675
Applied MSP Library(ies):	
Applied Taxonomy Tree:	Projects, Bruker Taxonomy

Rank (Quality)	Matched Pattern	Score Value	NCBI Identifier
1 (+++)	Pseudomonas savastanoi ssp savastanoi LMG 2209T HAM	2.316	29438
2 (++)	Pseudomonas savastanoi ssp savastanoi LMG 5011 HAM	2.136	29438
3 (++)	Pseudomonas caricapapayae LMG 2152T HAM	2.058	46678
4 (+)	Pseudomonas syringae ssp syringae DSM 6693 HAM	1.884	317
5 (+)	Pseudomonas congelans DSM 14939T HAM	1.884	200452
6 (+)	2502	1.815	451320
7 (+)	Pseudomonas syringae ssp syringae LMG 1247T HAM	1.814	317
8 (+)	2501	1.798	451320
9 (+)	Pseudomonas agarici DSM 11810T HAM	1.774	46677
10 (+)	Pseudomonas avellanae DSM 11809T DSM	1.734	46257
