# Supplementary material for: Identification and Characterisation of Pseudomonas savastanoi pv. savastanoi as the Causal Agent of Olive Knot Disease in Croatian, Slovenian and Portuguese Olive (Olea europaea L.) Orchards
Source: Plants (Basel). 2023 Jan 9;12(2):307. doi: 10.3390/plants12020307 (PMC9865541; doi:10.3390/plants12020307)
Supplement: Supplementary file 1 [file plants-12-00307-s001.zip › Table S1. Bacteria strains isolated from olive knots in Slovenia identified using mass spectrometry.pdf]

Table S1. Bacteria strains isolated from olive knots in Slovenia identified with MALDI-TOF mass spectrometry.

Isolate code	Olive variety	SV*	Bacterium name
F1-2	Frantoio	2.309	*Pseudomonas savastanoi* pv. *savastanoi*
F1-3	Frantoio	2.289	*Pseudomonas savastanoi* pv. *savastanoi*
F1-4	Frantoio	2.257	*Pseudomonas savastanoi* pv. *savastanoi*
F1-5	Frantoio	2.337	*Pseudomonas savastanoi* pv. *savastanoi*
F1-6	Frantoio	2.334	*Pseudomonas savastanoi* pv. *savastanoi*
A1-1	Arbequina	2.378	*Pseudomonas savastanoi* pv. *savastanoi*
A1-3	Arbequina	2.316	*Pseudomonas savastanoi* pv. *savastanoi*
A1-4	Arbequina	2.325	*Pseudomonas savastanoi* pv. *savastanoi*
A1-5	Arbequina	2.263	*Pseudomonas savastanoi* pv. *savastanoi*
L2-1	Leccino	2.293	*Pseudomonas savastanoi* pv. *savastanoi*
L2-2	Leccino	2.288	*Pseudomonas savastanoi* pv. *savastanoi*
L2-3	Leccino	2.370	*Pseudomonas savastanoi* pv. *savastanoi*
L2-4	Leccino	2.315	*Pseudomonas savastanoi* pv. *savastanoi*
L2-5	Leccino	2.206	*Pseudomonas savastanoi* pv. *savastanoi*
L6-2	Leccino	2.293	*Pseudomonas savastanoi* pv. *savastanoi*
L6-3	Leccino	2.278	*Pseudomonas savastanoi* pv. *savastanoi*
L6-4	Leccino	2.335	*Pseudomonas savastanoi* pv. *savastanoi*
F3-2	Frantoio	2.238	*Pseudomonas savastanoi* pv. *savastanoi*
F3-3	Frantoio	2.294	*Pseudomonas savastanoi* pv. *savastanoi*
F3-4	Frantoio	2.370	*Pseudomonas savastanoi* pv. *savastanoi*
F3-5	Frantoio	2.243	*Pseudomonas savastanoi* pv. *savastanoi*
F3-1	Frantoio	2.267	*Pseudomonas savastanoi* pv. *savastanoi*
L1-1	Leccino	2.383	*Pseudomonas savastanoi* pv. *savastanoi*
L6-5	Leccino	2.308	*Pseudomonas savastanoi* pv. *savastanoi*
L5-3	Leccino	2.314	*Pseudomonas savastanoi* pv. *savastanoi*
L5-1	Leccino	2.289	*Pseudomonas savastanoi* pv. *savastanoi*
L5-5	Leccino	2.320	*Pseudomonas savastanoi* pv. *savastanoi*
L5-4	Leccino	2.257	*Pseudomonas savastanoi* pv. *savastanoi*
L5-2	Leccino	2.291	*Pseudomonas savastanoi* pv. *savastanoi*
F4-2	Frantoio	2.297	*Pseudomonas savastanoi* pv. *savastanoi*
F4-1	Frantoio	2.276	*Pseudomonas savastanoi* pv. *savastanoi*
F4-4	Frantoio	2.297	*Pseudomonas savastanoi* pv. *savastanoi*
F4-3	Frantoio	2.334	*Pseudomonas savastanoi* pv. *savastanoi*
F4-5	Frantoio	2.178	*Pseudomonas savastanoi* pv. *savastanoi*
L4-1	Leccino	2.291	*Pseudomonas savastanoi* pv. *savastanoi*
L4-2	Leccino	2.26	*Pseudomonas savastanoi* pv. *savastanoi*
L4-5	Leccino	<0	*No ID*
L4-4	Leccino	2.265	*Pseudomonas savastanoi* pv. *savastanoi*
L4-3	Leccino	2.256	*Pseudomonas savastanoi* pv. *savastanoi*
L6-1	Leccino	2.278	*Pseudomonas savastanoi* pv. *savastanoi*
F2-2	Frantoio	2.369	*Pseudomonas savastanoi* pv. *savastanoi*
F2-4	Frantoio	2.283	*Pseudomonas savastanoi* pv. *savastanoi*
F2-5	Frantoio	2.351	*Pseudomonas savastanoi* pv. *savastanoi*
F2-3	Frantoio	2.347	*Pseudomonas savastanoi* pv. *savastanoi*
F2-1	Frantoio	2.228	*Pseudomonas savastanoi* pv. *savastanoi*
At1-2	Ascolana tenera	2.357	*Pseudomonas savastanoi* pv. *savastanoi*
At1-5	Ascolana tenera	2.325	*Pseudomonas savastanoi* pv. *savastanoi*
At1-1	Ascolana tenera	2.321	*Pseudomonas savastanoi* pv. *savastanoi*

At1-4	Ascolana tenera	2.289	*Pseudomonas savastanoi* pv. *savastanoi*
I1-5	Itrana	1.599	*Curtobacterium flaccumfaciens* DSM 20149 DSM
At1-3	Ascolana tenera	2.361	*Pseudomonas savastanoi* pv. *savastanoi*
I1-4	Itrana	1.604	*Curtobacterium flaccumfaciens* DSM 20149 DSM
I1-3	Itrana	1.688	*Curtobacterium flaccumfaciens* DSM 20149 DSM
I1-2	Itrana	2.391	*Bacillus megaterium*
I1-1	Itrana	1.642	*Curtobacterium flaccumfaciens* DSM 20149 DSM
At2-6	Ascolana tenera	2.292	*Pseudomonas savastanoi* pv. *savastanoi*
At2-1	Ascolana tenera	2.304	*Pseudomonas savastanoi* pv. *savastanoi*
At2-3	Ascolana tenera	2.300	*Pseudomonas savastanoi* pv. *savastanoi*
At2-4	Ascolana tenera	2.244	*Pseudomonas savastanoi* pv. *savastanoi*
At2-5	Ascolana tenera	2.316	*Pseudomonas savastanoi* pv. *savastanoi*

*Score value (SV) means; 2.000–3.000: highly probable species identification, 1.700–1.999: secure genus identification, probable species identification; 0.000–1.699: not reliable identification.
