# Supplementary material for: Identification and Characterisation of Pseudomonas savastanoi pv. savastanoi as the Causal Agent of Olive Knot Disease in Croatian, Slovenian and Portuguese Olive (Olea europaea L.) Orchards
Source: Plants (Basel). 2023 Jan 9;12(2):307. doi: 10.3390/plants12020307 (PMC9865541; doi:10.3390/plants12020307)
Supplement: Supplementary file 1 [file plants-12-00307-s001.zip › Table S2. Characterisation of molecularly identified Pss isolates.pdf]

Table S2. Characterisation of molecularly identified *Pss* isolates from Slovenian, Croatian and Portuguese olive orchards*.

Country	Isolate code	Olive variety	Fluorescence on KB	Levan production	Oxidase activity	Pectolytic activity	Arginin dihydrolase activity	Tobacco hypersensitive reaction
Croatia	V4B	Buža	-	-	-	-	-	-
	V4P	Pendolino	+	-	-	-	-	-
	V7K	Karbonaca	-	-	-	-	-	+
	V9B	Buža	-	-	-	-	-	+
	R12L	Leccino	+	-	-	-	-	+
	P15TR	Italian rosinjola	+	-	-	-	-	+
	P15N	Nocciara	+	-	-	-	-	+
	B17L	Leccino	-	-	-	-	-	+
	B19F	Frantoio	+	-	-	-	-	+
	B19L	Leccino	-	-	-	-	-	+
	B19P	Pendolino	+	-	-	-	-	+
	B21F	Frantoio	+	-	-	-	-	+
	B21L	Leccino	-	-	-	-	-	+
	B23F	Frantoio	-	-	-	-	-	+
	16L	Leccino	+	-	-	-	-	+
	17L	Leccino	-	-	DP	-	-	+
	NIN	Unknown	-	-	-	-	-	+
	U26F	Frantoio	-	-	-	-	-	+
	U27L	Leccino	-	-	-	-	-	+
	VS28L	Leccino	-	-	DP	-	-	+
	VS28N	Unknown	-	-	DP	-	-	+
	VS29L	Leccino	-	-	DP	-	-	+
	VS31L	Leccino	-	-	-	-	-	+
	R34F	Frantoio	-	-	-	-	-	+
	BR37L	Leccino	-	-	-	-	-	+
	N40M	Maurino	-	-	-	-	-	+
	N41F	Frantoio	-	-	-	-	-	+
	N42F	Frantoio	-	-	-	-	-	+
	N43L	Leccino	-	-	-	-	-	+
	N43P	Pendolino	-	-	-	-	-	+
	P44F	Frantoio	-	-	+	-	-	+
	B45C-PR	Porečka rosulja	+	-	+	-	-	+
Slovenia	F1-2	Frantoio	+	-	DP	-	-	+
	F1-3	Frantoio	+	-	DP	-	-	+
	F1-5	Frantoio	+	-	DP	-	-	+
	A1-1	Arbequina	+	-	DP	-	-	+
	A1-3	Arbequina	+	-	DP	-	-	+
	A1-4	Arbequina	+	-	DP	-	-	+
	L2-1	Leccino	+	-	DP	-	-	+
	L2-3	Leccino	+	-	DP	-	-	+
	L6-2	Leccino	+	-	-	-	-	+
	L6-4	Leccino	+	-	DP	-	-	+
	F3-2	Frantoio	+	-	DP	-	-	+
	F3-4	Frantoio	+	-	DP	-	-	+
	F3-5	Frantoio	+	-	DP	-	-	+
	L1-1	Leccino	+	-	+	-	-	+
	L5-3	Leccino	+	-	DP	-	-	+

	L5-1	Leccino	+	-	-	-	-	+
	L5-4	Leccino	+	-	DP	-	-	+
	F4-2	Frantoio	+	-	-	-	-	+
	F4-1	Frantoio	+	-	+	-	-	+
	F4-4	Frantoio	+	-	+	-	-	+
	L4-1	Leccino	+	-	DP	-	-	+
	L4-4	Leccino	+	-	DP	-	-	+
	L6-1	Leccino	+	-	DP	-	-	+
	F2-2	Frantoio	+	-	DP	-	-	+
	F2-5	Frantoio	+	-	DP	-	-	+
	At1-2	Ascolana tenera	+	-	DP	-	-	+
	At1-5	Ascolana tenera	+	-	DP	-	-	+
	At1-4	Ascolana tenera	+	-	DP	-	-	+
	At2-6	Ascolana tenera	+	-	DP	-	-	+
	At2-4	Ascolana tenera	+	-	-	-	-	+
**Portugal**	PT15	Cordovil	+	-	-	-	-	+
	PT17	Madural	+	-	-	-	-	+

*+ = positive; - = negative; DP = delayed positive.
